# The thiostrepton A tryptophan methyltransferase TsrM catalyses a cob(II)alamin-dependent methyl transfer reaction

**DOI:** 10.1038/ncomms9377

**Published:** 2015-10-12

**Authors:** Alhosna Benjdia, Stéphane Pierre, Carmen Gherasim, Alain Guillot, Manon Carmona, Patricia Amara, Ruma Banerjee, Olivier Berteau

**Affiliations:** 1 INRA, ChemSyBio, UMR 1319 Micalis, F-78350 Jouy-en-Josas, France; 2 AgroParisTech, ChemSyBio, UMR Micalis, F-78350 Jouy-en-Josas, France; 3 Department of Biological Chemistry, University of Michigan Medical School, Ann Arbor, Michigan 48109-0600, USA; 4 Metalloproteins Unit, Institut de Biologie Structurale, UMR5075, CEA, CNRS, Univ. Grenoble-Alpes. 71, Avenue des Martyrs, CS 10090, 38044 Grenoble, France

## Abstract

Ribosomally synthesized and post-translationally modified peptides (RiPPs) are a novel class of natural products including several antibiotics and bacterial toxins. In countless RiPP biosynthetic pathways, cobalamin-dependent radical SAM (B_12_/rSAM) enzymes play a pivotal role. In the biosynthetic pathway of the antibiotic and anti-cancer agent thiostrepton A, TsrM, a B_12_/rSAM enzyme, catalyses the transfer of a methyl group to an electrophilic carbon atom of tryptophan. Here we show that methylcob(III)alamin is the probable physiological enzyme cofactor, and cob(II)alamin rather than cob(I)alamin is a key reaction intermediate. Furthermore, we establish that TsrM and a triple-alanine mutant alkylate cob(II)alamin efficiently leading to the synthesis of MeCbl. Exploiting TsrM substrate ambiguity, we demonstrate that TsrM does not catalyse substrate H-atom abstraction like most radical SAM enzymes. Based on these data, we propose an unprecedented radical-based C-methylation mechanism, which further expands the chemical versatility of rSAM enzymes.

## Abstract (web summary)

Cobalamin-dependent radical SAM enzymes are proposed as methyltransferases in many biosynthetic pathways. Here, the authors study a pathway involving the methylation of tryptophan, showing that methylcob(III)alamin is the most likely cofactor and propose a radical-based C-methylation mechanism.

## Graphical abstract

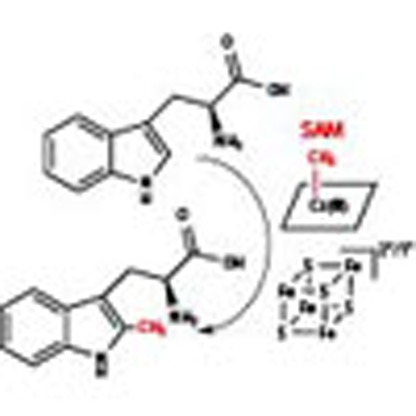

Cobalamin-dependent radical *S*-adenosyl-L-methionine (B_12_/rSAM) enzymes are characterized by the unique association of an *N*-terminal B_12_-binding domain and a rSAM-domain containing three critical cysteine residues presumed to be involved in coordination of a [4Fe-4S] centre^1^. These enzymes have been identified as potential methyltransferases in the biosynthetic pathways of many pharmaceutically relevant compounds previously believed to be produced by either non-ribosomal peptide synthetases or polyketide synthases. For example, the macrocyclic thiopeptide thiostrepton A^2^, the cytotoxic peptide polytheonamide A^3^ and the antibiotic bottromycin A^4^ have been shown recently to originate from ribosomally synthesized peptides undergoing extensive and unconventional post-translational modifications notably *C*-methylations catalysed by rSAM enzymes. These natural products are now described by the acronym RiPP^5^. In addition to RiPPs, many other natural products require B_12_/rSAM enzymes for their biosynthesis^6^, as recently demonstrated for the aminoglycoside antibiotic, gentamicin^7^. Despite the major importance of the emerging B_12_/rSAM family of enzymes, only few members have been investigated thus far and their mechanism remains very poorly understood.

Thiostrepton A biosynthesis is a complex process, which involves the formation of a quinaldic moiety from tryptophan (Fig. 1). The first step in the quinaldic ring expansion is an unusual methyl transfer to tryptophan C2 (refs 8, 9), the most electrophilic carbon atom in the indole ring^10^. We recently identified TsrM as the B_12_/rSAM enzyme responsible for this unconventional methyl transfer reaction and demonstrated that its activity is dependent on *S*-adenosyl-L-methionine (SAM) and the presence of a cobalamin derivative^1^. More recently a novel B_12_/rSAM enzyme, GenK, a key enzyme in the gentamicin biosynthetic pathway, was shown to catalyse methyl transfer to an unactivated carbon with the concomitant production of 5′-deoxyadenosine (5′-dA)^7^. This latter result demonstrated that GenK belongs to the superfamily of radical SAM enzymes and suggested that GenK, like most other rSAM enzymes investigated so far, produces a 5′-deoxyadenosyl radical (5′-dA·) for the radical activation of its substrate^11^. Consistent with this hypothesis, and in sharp contrast to our findings on TsrM^1^, the activity of GenK was shown to be dependent on the presence of an external electron donor. To gain further insight into the mechanism of B_12_/rSAM methyltransferases, we have investigated TsrM mechanism in detail.

Our study establishes that methylcob(III)alamin (MeCbl) is the probable enzyme cofactor. It also supports cob(II)alamin as a key reaction intermediate and that H-atom abstraction from the substrate does not occur during catalysis, making the chemistry of the TsrM-catalysed reaction unique. We further demonstrate that TsrM produces MeCbl from cob(II)alamin and that artificially generated cob(I)alamin does not enhance enzyme activity, contrary to what has been shown with previously investigated MeCbl-dependent enzymes^12^. Finally, TsrM exhibits high turnover and a broad substrate ambiguity unlike other radical SAM enzymes investigated to date. These data are consistent with TsrM making unique use of MeCbl and cob(II)alamin being a central intermediates during catalysis. We propose an unprecedented mechanism for enzymatic *C*-methylation that explains how TsrM, and possibly other B_12_/rSAM enzymes, catalyses efficient methyl transfer to an unsaturated electrophilic carbon atom in RiPPs biosynthesis.

## Results

### Identification of the physiological B_12_ cofactor of TsrM

We, and others, have reported that B_12_/rSAM enzymes can catalyse efficient methyl transfer in the presence of MeCbl but also in the presence of other B_12_ analogues, hydroxycob(III)alamin (OHCbl) or cyanocob(III)alamin^1^,^7^. We further assessed the ability of 5′-deoxyadenosylcob(III)alamin (AdoCbl) to support TsrM activity. As shown, AdoCbl could substitute for MeCbl in the TsrM reaction albeit only in the presence of light (Fig. 2a). High-performance liquid chromatography (HPLC) analysis revealed that under the anaerobic turnover conditions, OHCbl was formed likely resulting from the oxidation of cob(II)alamin produced by the photolysis of AdoCbl^13^ or MeCbl. Thus, the HPLC profiles of the reaction mixtures containing MeCbl or AdoCbl were virtually identical showing total conversion of AdoCbl or MeCbl to OHCbl with the formation of Me-Trp and SAH (Fig. 2a and Supplementary Fig. 1).

To prevent photolysis, TsrM assays were repeated in the dark. Under these conditions, both alkylcobalamins were stable for several hours and formation of the Me-Trp and SAH products was observed only in the presence of MeCbl or OHCbl (Fig. 2a,b). Under these assay conditions, the specific activity of TsrM was 3.9±0.2 μmol min^−1^ mg^−1^ with MeCbl and 3.5±0.2 μmol min^−1^ mg^−1^ with OHCbl reduced to cob(II)alamin by dithiothreitol (DTT), whereas no activity was observed with AdoCbl (Fig. 2b). Increasing the concentration of DTT to 12 mM, in the presence of OHCbl, increased the specific activity up to 4.8±0.3 μmol min^−1^ mg^−1^. In contrast, the activity was reduced to 0.86±0.1 μmol min^−1^ mg^−1^ when 1 mM DTT was present in the reaction with the appearance of a lag phase probably associated with reduction of OHCbl and/or the enzyme [Fe-S] centre (Fig. 2c).

Substoichiometric concentrations of MeCbl, as low as 1 μM, were sufficient to support efficient methyl transfer (1.5±0.1 μmol min^−1^ mg^−1^) consistent with MeCbl functioning as an enzyme cofactor and not a substrate (Supplementary Fig. 2). Furthermore, this result indicated that only a portion of the protein had been efficiently reconstituted in an active form. In each assay condition, the amount of Me-Trp strictly correlated with the amount of SAH produced consistent with SAM being the source of the methyl group (Fig. 2b,c).

To determine whether cobalt coordination by the endogenous dimethylbenzimidazole ligand is required for TsrM activity, MeCbl was substituted with the methylcobinamide (MeCbi) derivative^14^. Although MeCbi supported enzyme activity, it was 5±0.3% as effective as MeCbl (Fig. 3), demonstrating the importance of the nucleotide tail.

Collectively, these results demonstrate that only MeCbl supports turnover when photolysis (of AdoCbl) or reduction (of OHCbl) are prevented. Importantly, the specific activity of TsrM in the presence of MeCbl and cob(II)alamin (generated by the anaerobic reduction of OHCbl), are identical, consistent with a role of cob(II)alamin in the catalytic cycle.

### Cob(II)alamin is a central reaction intermediate

As MeCbl is the likely proximal methyl group donor to Trp, in the presence of deuterated SAM (*d*_*3*_-SAM) and an excess of unlabelled MeCbl, the formation of CH_3_-Trp along with CD_3_-Trp would be expected. We analysed the reactions performed in the presence of MeCbl and protected from light by using high-resolution liquid chromatography–mass spectrometry (LC-MS). Under these conditions, which preserved MeCbl, we detected the formation of 1.5±1% of CH_3_-Trp (*m/z*=219.11) along with 98±2% CD_3_-Trp (*m/z*=222.13). Analysis of the *d*_*3*_-SAM used in the assay showed a CH_3_/CD_3_ ratio of 1.3±0.1%, similar to the one measured for Me-Trp. Furthermore, if we used OHCbl instead of MeCbl, in the presence of *d*_*3*_-SAM, the CH_3_/CD_3_-Trp ratio measured was in the same range (2.3±1%), demonstrating that free MeCbl does not significantly influence Me-Trp labelling. These results are consistent with the use of SAM as the exclusive methyl donor by TsrM, even in the presence of a large excess of free MeCbl.

As shown above, with the exception of MeCbl, all other cobalamin derivatives had to be converted to cob(II)alamin to support catalysis (that is, after the facile reduction of OHCbl by DTT or photolysis of alkylcobalamins). Ultraviolet–visible spectroscopic monitoring of the reaction in the presence of MeCbl in the dark and under anaerobic and reducing conditions showed no evidence for the formation of cob(I)alamin (Fig. 4). In contrast, we measured a weak spectral shift from ∼520 to ∼476 nm with an increase in absorbance between 400 and 476 nm characteristic of the formation of cob(II)alamin (Fig. 4b). This spectral shift occurred during the first 30 min of the reaction, under steady-state conditions, and no further changes were observed over the next 60 min.

As cob(I)alamin is a well-known reaction intermediate in cobalamin-dependent methyl-transferases^15^, we measured its effect on the reaction. Cob(I)alamin can be generated using titanium citrate (TiC) as a reducing agent^16^. Furthermore, TiC has been demonstrated to be a potent activator of cobalamin-dependent methyltransferases containing inactive cob(II)alamin^14^.

TsrM was assayed under anaerobic and reducing conditions, in the presence of 3 mM TiC and 200 μM of OHCbl. As shown in Fig. 5, immediately after addition of TiC, MeCbl production was evident ([M+2H]^2+^=674.3) and OHCbl conversion was complete after few minutes (Fig. 5a,b and Supplementary Fig. 3). After 2 h, the amount of Me-Trp produced by TsrM was 8.0±0.2% of that produced in the absence of TiC indicating enzyme inhibition (Fig. 5 and Supplementary Fig. 3). Decreasing the concentration of TiC to 150 μM restored enzyme activity and allowed the *in situ* production of MeCbl (∼2 μM). However, similar amount of Me-Trp was formed (91±6%) compared with the reaction performed in the absence of TiC (Fig. 5a,b).

If TiC allowed, after reduction, the efficient conversion of OHCbl into MeCbl, low amounts of MeCbl could also be detected by LC-MS in the reaction performed with TsrM and in the absence of TiC (Fig. 5c). In contrast, the control reaction, performed in the absence of TsrM and TiC, showed no evidence of MeCbl.

We repeated the enzymatic reaction without TiC but with a higher concentration of OHCbl (1 mM) to favour the displacement to MeCbl. The reaction, protected from light, was analysed at the end of the steady state. As shown, we succeeded to slightly increase the amount of MeCbl produced by TsrM (Fig. 6a,b). To ascertain, whether or not, MeCbl was enzymatically produced, we performed two control reactions, one lacking TsrM and the other one with a mutant enzyme generated by substituting the three conserved cysteine residues present in the radical SAM motif (CxxxCxxC) by alanine residues. As shown in Fig. 6, in the absence of enzyme, no MeCbl could be detected even after 12 h of incubations (Fig. 6a,c). Further increasing the amount of DTT, iron and sulphide up to ten times, in the absence of enzyme, did not support the formation of MeCbl under our assay conditions either (Supplementary Fig. 4). In the presence of TsrM, MeCbl was easily identified after 2 h of reaction (Fig. 6c). Surprisingly, in the reaction performed with the AxxxAxxA mutant which does not contains measurable amounts of iron, MeCbl was produced very efficiently (Fig. 6a,c) and accumulated over time.

The level of SAH produced by TsrM was maximum at 2 h (∼1 mM) and correlated with the amount of Me-Trp, whereas the amount of SAH produced by the mutant was maximum at 12 h (∼300 μM) and correlated with the amount of MeCbl produced, demonstrating that TsrM catalyses the alkylation of cob(II)alamin (Fig. 6a,d). Further investigations revealed that the production of MeCbl by the mutant was strictly dependent on OHCbl reduction by DTT.

### TsrM does not abstract a substrate H-atom

Recent work on GenK, another B_12_/rSAM enzyme, revealed that 5′-dA is generated during the reaction, suggesting the formation of a transient 5′-dA· for substrate H-atom abstraction^7^, as seen with most rSAM enzymes^17^,^18^,^19^,^20^,^21^,^22^. With TsrM, despite the high turnover numbers and the sensitivity of the LC-MS method used, evidence for 5′-dA formation was not obtained under any experimental conditions. Furthermore, the amount of SAH correlated, under all assays conditions, with the amount of Me-Trp, supporting the use of SAM exclusively as a methyl donor in the reaction.

To further demonstrate that TsrM does not abstract a H-atom from its substrate, fully deuterated Trp (^15^N_2_, D_*8*_-Trp) was used to track the non-exchangeable H-atoms, notably the β H-atoms, which are the logical target for abstraction by a putative 5′-dA· (ref. 23). We also considered the exchangeable H-atoms on the amino- and carboxy-groups and the N1 of the indole ring as potential targets (Fig. 7). Accordingly, TsrM was incubated with *d*_3_-SAM and different substrates (that is, ^15^N_2_, D_*8*_-Trp (**3**), NMα-Trp (**5**) or NM1-Trp (**7**)) and the reactions were analysed by HPLC coupled to fluorescence detection and LC-MS. When **3** was used as a substrate, a 16-Da mass increase was observed corresponding to the transfer of a -CD_3_ group (+18 Da) and the loss of a deuterium atom (−2 Da; Fig. 7a, Supplementary Figs 5–9 and Supplementary Table 1). These data establish unequivocally that, among the non-exchangeable Trp H-atoms, only the C2 H-atom is lost during the reaction.

Interestingly, although NMα-Trp showed 28% substrate efficiency as compared with Trp (Fig. 7 and Supplementary Fig. 8), NM1-Trp was not a substrate (Fig. 7a and Supplementary Fig. 9), which indicates that TsrM is unable to either bind NM1-Trp or catalyse the transfer reaction. To distinguish between these two alternatives, we incubated TsrM with a 1:1 mixture of Trp and NM1-Trp. As shown in Fig. 7b,c, in the presence of NM1-Trp, the methyl transfer activity of TsrM was inhibited (from 6.8 μmol min^−1^ to 0.5 μmol min^−1^) indicating that TsrM binds to NM1-Trp.

### Substrate ambiguity of TsrM

To further probe the involvement of the exchangeable H-atoms in the reaction, we evaluated the activity of TsrM with 11 substrate derivatives (Fig. 8a, Supplementary Figs 10,11 and Supplementary Table 2). TsrM catalysed methyl transfer to all Trp derivatives substituted on the indole ring. Of interest, TsrM had a specific activity of 1.3, 1.2 and 0.6 μmol min^−1^ mg^−1^ with compounds substituted at C5 (that is, 5-Me-, 5-OH- and 5-F-Trp (compounds **11**, **13** and **15**, respectively)). This result is in contrast with the electrophilic substitution catalysed by some tryptophan prenyltransferases, which are highly sensitive to the presence of activating and deactivating groups^24^, but is consistent with a radical addition to Trp. TsrM activity was substantially lower (∼10%) when L-Trp was substituted by D-Trp **9** or its derivative, 6-fluoro-L/D-Trp **17**, underscoring the importance of the stereochemistry at the α-carbon (Supplementary Table 2).

When assaying TsrM with either melatonin or 3-methylindole, both of which lack the α–carboxy group of Trp (compounds **23** and **19**, Fig. 8), the activity of TsrM dropped to <20% (Fig. 8c and Supplementary Table 2). The absence of intrinsic charges in **19**, likely contributed to our inability to characterize the product formed by mass spectrometry. We thus assayed serotonin as a potential substrate (compound **21**, Fig. 8). TsrM showed higher efficiency with **21** and mass spectrometric analysis confirmed the identity of the methyl-serotonin product formed (compound **22**, Fig. 8c). Collectively, these data show that substitutions on the indole ring have little influence on TsrM activity and that the presence of the amino- and carboxyl groups of Trp, although important, is not essential for this promiscuous methylating enzyme. These results are in sharp contrast with those reported for other rSAM enzymes catalysing tyrosine^21^ modification such as ThiH^25^.

Altogether, these results demonstrate that, except for the H-atom at C2, none of the other exchangeable or non-exchangeable H-atoms of Trp are directly involved in catalysis.

## Discussion

**Figure 1:**
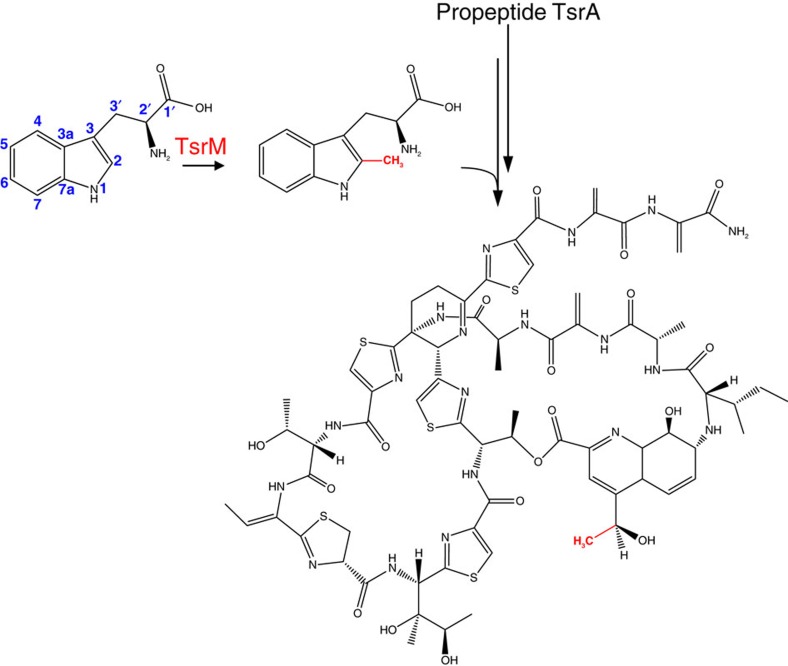
Reaction catalysed by TsrM in the thiostrepton A biosynthetic pathway. TsrM catalyses methyl transfer to the C2 position of tryptophan. Methyl-tryptophan then serves as a precursor of the quinaldic moiety incorporated into the modified propeptide TsrA leading to the biosynthesis of thiostrepton A. (The methyl group transferred by TsrM is shown in red).

**Figure 2:**
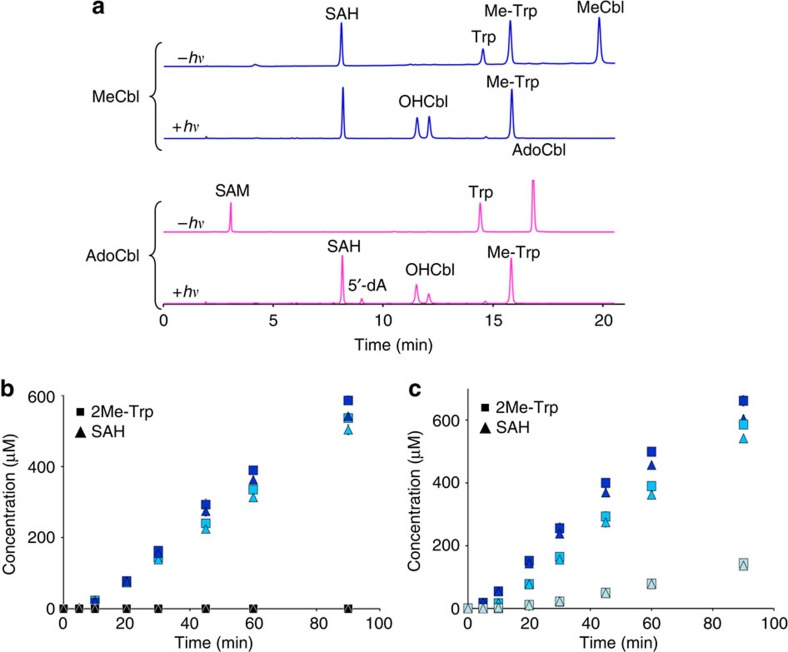
*In vitro* activity of TsrM. (**a**) HPLC analysis of TsrM reactions in the presence of MeCbl (blue traces) or AdoCbl (pink traces) with (+*hν*) or without (-*hν*) light exposure (ultraviolet detection at 278 nm). TsrM was incubated for 12 h in the presence of Trp, SAM and MeCbl or AdoCbl under anaerobic and reducing conditions (see Supporting Information for details). In the dark, both MeCbl and AdoCbl (traces "-*hν*") were stable for hours, whereas they are rapidly photolyzed to cob(II)alamin in the light and subsequently oxidized to OHCbl during analysis (traces “+*hν*”). As shown, in the absence of light, only MeCbl supports the formation of Me-Trp. (**b**) Time course for the production of 2-Me-Trp (▪) and SAH (▴) by TsrM in the dark in the presence of MeCbl (dark blue symbols), OHCbl (cyan symbols) or AdoCbl (black symbols). TsrM (23 μM) was incubated under anaerobic and reducing conditions in the presence 1 mM SAM, 1 mM Trp and 6 mM DTT. Time points are the average of two independent experiments. (**c**) Time course for the production of 2-Me-Trp (▪) and SAH (▴) by TsrM in the presence of OHCbl and increasing amounts of DTT. TsrM (23 μM) was incubated under anaerobic and reducing conditions in the presence of 1 mM SAM, 1 mM Trp and 1, 6 or 12 mM DTT (light blue, cyan and dark blue, respectively). Time points are the average of two independent measurements.

**Figure 3:**
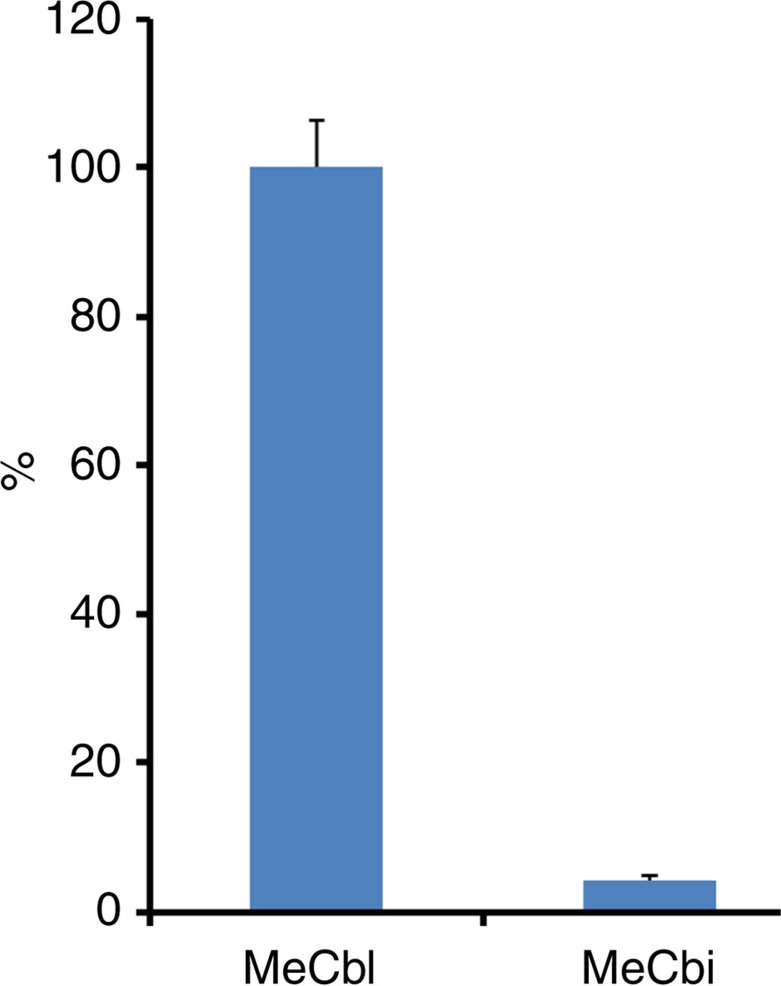
Relative production of Me-Trp by TsrM in the presence of MeCbl or MeCbi. TsrM (20 μM) was incubated under anaerobic conditions in the presence of 6 mM DTT, SAM (1 mM), Trp (1 mM) and MeCbl or MeCbi (500 μM). Error bars indicate s.d.

The B_12_/rSAM family of enzyme is emerging as a major group of enzymes involved in key methylation reactions. Remarkably, they are common in the biosynthetic pathways of post-translationally modified peptides, the so-called RiPPs^5^, and in the biosynthesis of many natural products including major antibiotics. TsrM, a member of the B_12_/rSAM family of enzymes, is involved in the biosynthesis of the antibiotic thiostrepton A, and catalyses a methyl group transfer from SAM to the C2 of Trp during the first step of the quinaldic moiety ring expansion^1^ (Fig. 1). We have shown that, unlike the vast majority of rSAM enzymes, 5′-dA is not produced and an external electron donor is not required for catalysis. In contrast, GenK, another B_12_/rSAM enzyme, has recently been shown to produce 5′-dA during catalysis^7^. Although it was not determined whether the presumed 5′-dA· radical actually abstracts a substrate H-atom, the postulated reaction mechanism invokes consumption of two moles of SAM per turnover, one for the production of the 5′-dA· radical and the second for the transfer of a methyl group to its substrate (gentamicin X_2_) by an undefined mechanism.

In the present study, we demonstrate that in addition to MeCbl and MeCbi, efficient enzyme turnover can be achieved in the presence of cob(II)alamin generated either by photolysis of AdoCbl or by reduction of OHCbl. When we inhibited formation of cob(II)alamin from AdoCbl by performing the reactions in the dark, only MeCbl and MeCbi were competent, although MeCbl was by far the more efficient cofactor highlighting the importance of the dimethylbenzimidazole moiety. Furthermore, we establish here that the role of MeCbl is of a cofactor and not a substrate.

**Figure 4:**
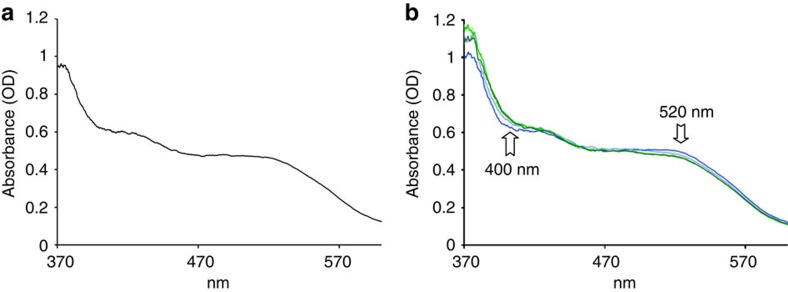
Ultraviolet–visible analysis of TsrM reaction in the dark. (**a**) Ultraviolet–visible spectrum of TsrM after enzyme reconstitution and addition of a twofold excess of MeCbl. (**b**) Ultraviolet–visible spectra of TsrM after addition of SAM and Trp (spectra were recorded from 0 to 90 min, from blue to dark green, respectively). After the addition of the substrates, a slight increase between 400 and 476 nm and decrease at 520 nm were observed during the first 30 min. These spectral changes are consistent with the formation of cob(II)alamin. No further modifications of the spectrum were measured during the next 60 min. OD, optical density.

Spectroscopic monitoring of the reaction in the dark allowed direct detection of cob(II)alamin under steady-state turnover conditions (Fig. 4). Surprisingly, and in contrast to what has been reported for cobalamin-dependent methyltransferases, we did not detect cob(I)alamin during turnover, which would indicate a nucleophilic transfer of the methyl group^15^.

**Figure 5:**
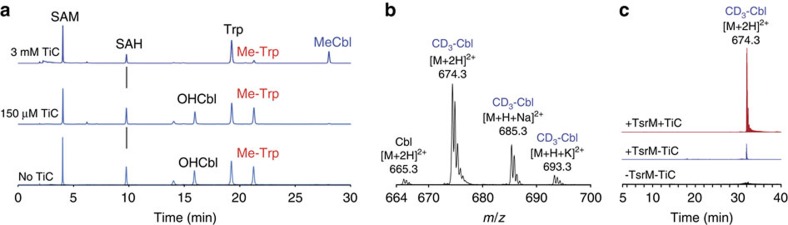
*In vitro* activity of TsrM in the presence of titanium citrate. (**a**) HPLC analysis of reactions performed with TsrM. TsrM (23 μM) was incubated under anaerobic conditions in the presence of DTT (3 mM), *d*_*3*_-SAM (1 mM), Trp (1 mM), OHCbl (200 μM) and various amounts of TiC (0, 150μM or 3 mM from bottom to top) for 2 h. HPLC analysis was performed on a C18 column with ultraviolet detection at 278 nm. OHCbl, after anaerobic reduction to cob(II)alamin by DTT and further reduction by TiC to cob(I)alamin, is converted into MeCbl. Maximal TsrM activity was monitored in the titanium citrate-free sample. (**b**) MS analysis of MeCbl produced in the reaction (theoretical mass for CD_3_-Cbl [M+2H]^2+^: 674.311). (**c**) Extracted-ion chromatogram of MeCbl produced in reactions ([M+2H]^2+^: 674.311). Reactions containing OHCbl (200 μM), DTT (3 mM), *d3*-SAM (1 mM) and Trp (1 mM) were incubated in the presence or absence of TsM and with or without TiC (3 mM). Reactions were analysed by HPLC performed on a C18 column. Chromatograms are normalized to the same intensity.

**Figure 6:**
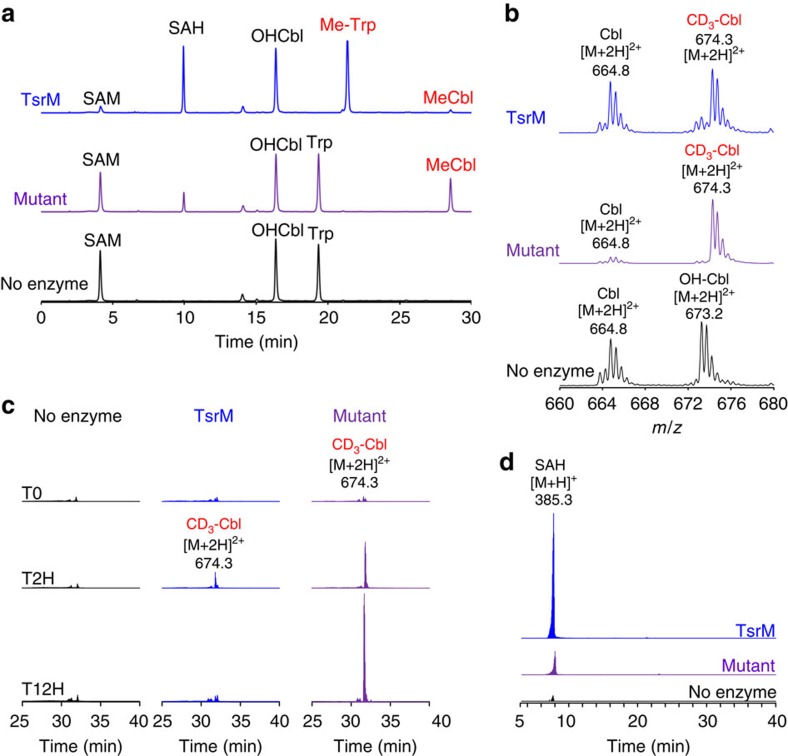
*In vitro* activity of TsrM and a triple-alanine mutant in the radical SAM motif. (**a**) HPLC analysis of the reactions performed with TsrM or a Cys→Ala mutant enzyme (Mutant). Reaction mixtures containing: DTT (3 mM), *d3-*SAM (1 mM), Trp (1 mM) and OHCbl (1 mM) were incubated for 12 h with TsrM (upper trace), a mutant enzyme containing a triple alanine substitution in the radical SAM CxxxCxxC motif (middle trace) or in the absence of enzyme (lower trace). HPLC analysis was performed on a C18 column with ultraviolet detection at 278 nm. (**b**) MS analysis of cobalamin derivatives produced in the reaction with TsrM (upper trace), the AxxxAxxA mutant (middle trace) or in the absence of enzyme (lower trace) (theoretical mass for CD_3_-Cbl [M+2H]^2+^: 674.31). (**c**) Extracted-ion chromatogram of MeCbl produced in reactions ([M+2H]^2+^: 674.311). Reactions containing OHCbl (1 mM), DTT (3 mM), *d3*-SAM (1 mM) and Trp (1 mM) were incubated without enzyme (no enzyme, left panel), with TsrM (TsrM, middle panel) or in the presence of a mutated enzyme in the radical SAM motif (that is, AxxxAxxA mutant; mutant, right panel). Each spectrum was normalized to the same intensity. (**d**) Extracted-ion chromatogram of SAH produced in the TsrM reaction ([M+H]^2+^: 385.31). Chromatograms are normalized to the same intensity. As shown, the mutant and wild-type enzymes were competent to produce SAH.

**Figure 7:**
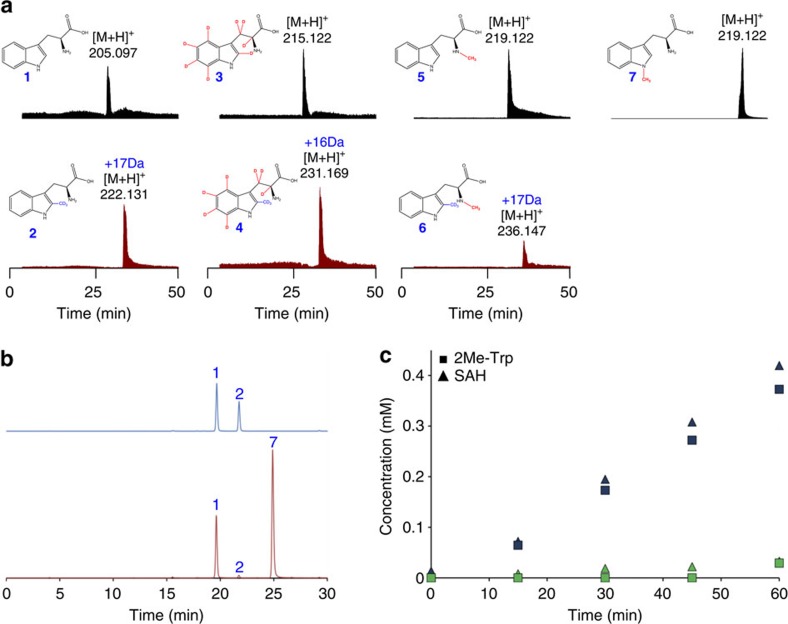
Activity of TsrM on deuterated and methylated tryptophan derivatives. (**a**) HPLC-MS analysis of TsrM incubated with Trp (**1**), (D_8_, ^15^N_2_) L-tryptophan (**3**), *N*M_α_-Trp (**5**) or NM_1_-Trp (**7**). The upper traces correspond to the *m/z* extracted-ion chromatograms of the substrates and the lower traces to the *m/z* extracted-ion chromatograms of the methylated products (that is, 2-CD_3_-Trp (**2**), 2-CD_3_-(D_7_, ^15^N_2_) L-tryptophan-Trp (**4**) and 2-CD_3_-*N*α-Me-L-Trp (**6**), respectively). Chromatograms were normalized according to the substrate signal (Supplementary Figs 5–8 for mass analysis and Supplementary Table 1 for assignment). (**b**) HPLC analysis of the reaction catalysed by TsrM monitored by fluorescence detection (Ex/Em=278/350 nm) after 2 h at 25 °C. TsrM was incubated in the presence of L-Trp (**1**) (upper trace) or **1** and NM_1_-Trp (**7**) (lower trace). As shown, in the presence of **7**, the synthesis of 2Me-Trp (**2**) is strongly inhibited. (**c**) Time course for production of Me-Trp and SAH by TsrM in the presence of Trp (blue symbols) or Trp and NM_1_-Trp (green symbols). TsrM (15 μM) was incubated under anaerobic conditions in the presence of 3 mM DTT with 1 mM MeCbl, 1 mM *d*_*3*_-SAM and 1 mM Trp or 1 mM Trp and NM_1_-Trp.

**Figure 8:**
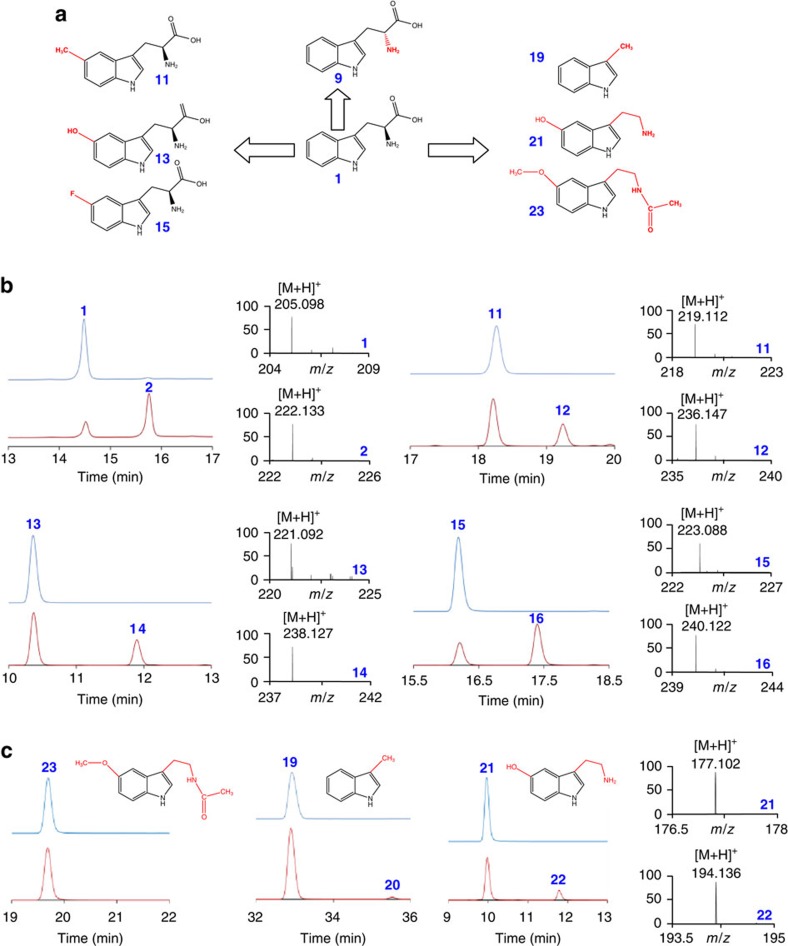
Substrate promiscuity of TsrM. (**a**) Structure of representative indole derivatives assayed as potential TsrM substrates (numbering as in Supplementary Table 2). (**b**) HPLC analysis of reactions performed with TsrM at T0 (upper traces) or after 12 h of incubation (lower traces) in the presence Trp (**1**), 5-Me-Trp (**11**), 5-OH-Trp (**13**) or 5- F-Trp (**15**) (fluorescence detection: Ex/Em=280/350 nm). The reactions were performed under anaerobic and reducing conditions in the presence of *d*_*3*_-SAM. Methyl transfer results in a +17 Da mass increase corresponding to the incorporation of CD_3_ and the formation of 2-Me-Trp (**2**), 2,5-Me-Trp (**12**), 2-Me, 5-OH-Trp (**14**) and 2-Me, 5-F-Tryptophan (**16**), respectively. (**c**) HPLC analysis of melatonin (**23**), 3-methyl indole (**19**) and serotonin (**21**) incubated in the presence of TsrM under anaerobic and reducing conditions in the presence of *d*_*3*_-SAM at T0 (upper traces) or after 12 h of incubation (lower traces). The mass spectra of serotonin and its product (22) are shown (right panel; see Supplementary Figs 10 and 11 for HPLC and MS analyses).

Following a previously established procedure^12^, we investigated the influence of TiC on the reaction. Indeed, this reductant is known to efficiently convert cob(II)alamin to cob(I)alamin, leading to the formation of MeCbl in the presence of a methyl donor (SAM) and to the activation of oxidatively inactivated cobalamin-dependent methyltransferases^15^. As shown (Fig. 5a), MeCbl was efficiently produced in the presence of TiC. Nevertheless, the generation of cob(I)alamin in the presence of 3 mM TiC inhibited the TsrM reaction, in contrast to what has been seen with cobalamin-dependent methyltransferases using an S_N_2-type mechanism^15^.

Quenching the reaction rapidly in the dark under anaerobic conditions, we were able to show that TsrM produces low amounts of MeCbl from cob(II)alamin. Interestingly, a mutant TsrM, lacking the canonical CxxxCxxC motif, was unable to catalyse methyl transfer to Trp, but produced large amounts of SAH and MeCbl. This result reveals the critical role of the [4Fe-4S] for methyl transfer to Trp and also demonstrates that TsrM can convert cob(II)alamin into MeCbl.

Although MeCbl is likely the proximal methyl group donor, significant incorporation of unlabelled methyl group into Trp was not observed despite the large excess of unlabelled MeCbl added over *d*_*3*_-SAM in the assays. This could be due to only a small proportion of the enzyme being active as suggested by the observation that 5 μM MeCbl was sufficient to obtain full activity with 20 μM enzyme. Alternatively, it is possible that the catalytic cycle is initiated in the cob(II)alamin state, requiring the loading of a methyl group on the corrin ring before the methyl transfer reaction to Trp.

Interestingly, we found that TsrM exhibits unusually high substrate ambiguity, the largest reported within the rSAM enzyme superfamily so far. Notably, TsrM is able to catalyse methyl group transfer to Trp derivatives lacking the carboxyl or the amino groups, the latter recently shown to be key for rSAM enzymes catalysing Tyr or Trp modifications^21^,^22^,^26^. The combined use of isotopically labelled Trp and various Trp derivatives revealed that only the C2 H-atom is replaced during the TsrM reaction and that, beside the indole ring, the remainder of the Trp is largely dispensable.

**Figure 9:**
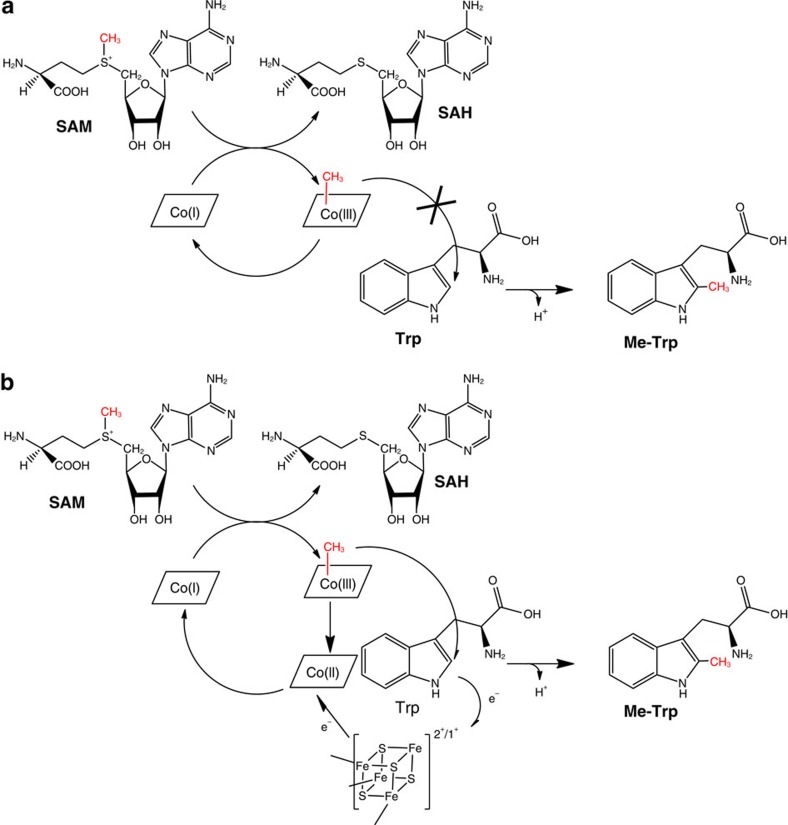
Mechanistic hypotheses and proposed mechanism for the methyl transfer reaction catalysed by TsrM. (**a**) S_N_2 alkylation hypothesis: Starting from MeCbl, a methyl group would be transferred directly to Trp with the exclusive formation of cob(I) and without the requirement of a [4Fe-4S] centre. This mechanistic hypothesis is similar to what have been reported for cobalamin-dependent methionine synthase. However, tryptophan is a much weaker nucleophile than the thiolate group found in the substrate of methionine synthase. This hypothesis appears thus unlikely. (**b**) Proposed mechanism for TsrM *C*-methylation reaction. Homolysis of the cobalt–carbon bond of MeCbl by TsrM produces a CH_3_· radical intermediate, which is transferred to the C2 position of Trp. Concomitant with Trp deprotonation, an electron is transferred to the [4Fe-4S]^2+^/^1+^ cluster, which reduces cob(II)alamin to cob(I)alamin. This supernucleophilic species can easily form MeCbl by an S_N_2 displacement of the methyl group of SAM-producing SAH. The double displacement of the methyl group during catalysis leads to a net configuration retention.

Based on our previous report^1^ and the current study, rather than a SN2 alkylation (Fig. 9a), we propose a novel mechanism for the TsrM-catalysed reaction in which the cobalt–carbon bond of MeCbl undergoes homolytic cleavage to generate a methyl radical (Fig. 9b). The Co–C bond dissociation energy in MeCbl is estimated to be 37 kcal mol^−1^, a value that is slightly higher than that for AdoCbl but still compatible with homolysis^27^. The calculated energy barriers for addition of a methyl radical to C2, C4, C5, C6 and C7, assuming that methyl group dissociation from MeCbl and addition to Trp are not concerted, are comparable to the experimental values determined for addition to ethene (6.7 kcal mol^−1^) and to benzene (8.9 kcal mol^−1^ (ref. 28); Supplementary Note 1 and Supplementary Tables 3 and 4). These values are too close to each other to be mechanistically discriminating, although addition to C2 versus the other carbon atoms is slightly favoured with a more stable radical species predicted to be formed.

Following transfer of the methyl radical to C2, deprotonation should occur, a process known to be spontaneous in some Trp-based radical processes^29^ and experimentally observed in the diheme enzyme MauG^30^. A one-electron transfer could then occur from the radical intermediate to the [4Fe-4S]^2+^ cluster and, then from the reduced [4Fe-4S]^1+^ cluster to cob(II)alamin forming cob(I)alamin as observed in the corrinoid iron-sulphur protein^31^,^32^. The cob(I)alamin thus generated is a supernucleophile and would attack the electrophilic methyl group of SAM to regenerate MeCbl and form SAH (Fig. 9b). Such nucleophilic displacement of the methyl group of SAM by cob(I)alamin is seen during the reactivation reaction catalysed by methionine synthase^15^. Interestingly, methionine synthase uses SAM as a methyl donor, albeit only during the reactivation cycle leading from cob(II)alamin to MeCbl, whereas the less reactive 5-methyltetrahydrofolate is the methyl donor in the catalytic cycle.

In addition to TsrM, we posit that GenK is likely to generate cob(II)alamin as an intermediate. Indeed, both enzymes have been shown to use OHCbl efficently in the presence of a reductant. Although in the presence of iron and a reducing agent reductive methylation of cob(II)alamin to MeCbl can occur, we have shown here that this did not occur under our assay conditions and that production of MeCbl from cob(II)alamin occurred primarily during TsrM turnover.

In seminal studies on thiostrepthon A biosynthesis, it has been shown that the methyl group present in the quinaldic part is transferred with a net retention of configuration^9^. Although fast tumbling of the methyl radical is expected in solution and should lead to a scrambling of the H-atoms, recent investigations have shown that methyl radicals originating from MeCbl do not behave like free methyl radicals^33^. Indeed, they have larger dynamic time scales due to geminate recombination and diffusive separation of the radical pair, which inhibit tumbling and could account for a net retention of configuration of the methyl group as determined previously^9^. Furthermore, cobalamin-dependent isomerases are known to exert exquisite control over radical trajectories during stereospecific group migrations^34^,^35^. It is thus likely that the methyl radical, similar to what has been extensively reported with the 5′-deoxyadenosyl radical, would not exist as a free species^36^. Furthermore, as the C2 of Trp is a weak nucleophile, it seems likely that a radical mechanism leading to the facile radical addition to Trp, would be favoured over a nucleophilic displacement one (Fig. 9b).

Finally, our mechanistic hypothesis is also supported by the recent discovery of new synthetic catalysts, which have been shown to efficiently and selectively methylate Trp at the C2 position by a radical-based process^37^, in contrast with an S_N_2-type mechanism, which would required the chemically challenging formation of a 4,7-dihydro intermediate^38^.

The methylation mechanism described here is unprecedented in biology and differs from the currently proposed one for GenK. It is possible that the B_12_/rSAM enzymes deploy different reaction mechanisms (that is, 5′-dA·-dependent or -independent), depending on the nature of the carbon atom (that is saturated unactivated *versus* unsaturated electrophilic) to which the methyl group is transferred. Detailed analysis of other members in the B_12_/rSAM family will be fundamental to understanding the range of chemically challenging reaction mechanisms they catalyse in seminal natural product synthetic pathways.

## Methods

### TsrM expression and purification

*Escherichia coli* BL21 (DE3) star (Life Technologies) transformed with an expressing plasmid (pASK-TsrM^1^), were grown at 37 °C in LB medium (9 l) supplemented with ampicillin (100 μg ml^−1^) and bacterial growth proceeded at 37 °C until the OD_600_ reached 0.6. Protein expression was induced by adding anhydrotetracycline (0.2 μg ml^−1^ final concentration) and medium was supplemented with 250 μM of iron. After overnight growth at 18 °C, the cells were collected by centrifugation at 5,000*g* for 15 min. The cells were suspended in 15 ml of buffer (50 mM Tris, 300 mM KCl, 10 mM MgCl_2_, 0.5 mM NaCl, 10% glycerol, pH 7.5) and disrupted by sonication on ice after adding 500 μl of Triton X-100 and 100 μl of 2-mercaptoethanol. Lysed cells were centrifuged at 45,000*g* at 4 °C for 1.5 h. The supernatant obtained was loaded onto a Streptactin high capacity (IBA GmbH) column previously equilibrated with the same buffer. The column was washed with 5 column volumes. Proteins were eluted with 6 ml of buffer containing desthiobiotine (0.6 mg ml^−1^) and DTT (3 mM) and, further concentrated with Amicon concentrator (Millipore) with a molecular cutoff of 10 kDa. The purified protein was stored at −80 °C and the purity was assayed on a 12% SDS–PAGE.

### Methyl-transferase activity

All the *in vitro* reactions were carried out in an anaerobic chamber in TsrM suspended with a buffer made of 50 mM Tris, 300 mM KCl, pH 7.5. The reaction mixtures were composed of the following compounds depending on the reactions: TsrM (20 μM), substrate (1 mM), SAM (1 mM), methyl-cobalamin or other cobalamin derivatives and DTT. Before incubation, TsrM was reconstituted by adding 3 mM of DTT at 12 °C during 15 min then Na_2_S and (NH_4_)_2_Fe(SO_4_)_2_ (500 μM) were added and the solution incubated at 12 °C during 4 h. Incubations were performed at 25 °C under strict anaerobic conditions.

### HPLC analysis

Samples were diluted in trifluoroacetic acid solution (TFA 0.1%). HPLC analysis was carried out on an Agilent 1200 series infinity with a reversed phase column (LiChroCART RP-18e 5 μm; Merck Millipore). The column was equilibrated with 100% solvent A (H_2_O, 0.1% TFA) and the following gradient was applied with the solvent B (80% CH_3_CN, 19.9% H_2_O, 0.1% TFA): 0–1 min 100% A/0% B; 1–22.5 min, a linear gradient up to 60% A/45% B; 22.5–27 min, a linear gradient to 100% A/0% B. Flow rate was 1 ml min^−1^ and detection was made at 257 and 278 nm with a diode array detector or by fluorescence detector (excitation at 278 nm and emission at 350 nm).

### Liquid chromatography–mass spectrometry/mass spectrometry analysis

Liquid chromatography–mass spectrometry/mass spectrometry analysis was performed using an Ultimate 3000 LC system (Dionex) connected to a LTQ-Orbitrap Discovery mass spectrometer (ThermoFisher) with a nanoelectrospray ion source or using a LTQ mass spectrometer (ThermoFisher) with a nanoelectrospray ion source in positive mode. Each reaction mixture was diluted ten times and 4 μl were loaded at a flow rate of 20 μl min^−1^ onto precolumn Pepmap C18 (0.3 by 5 mm, 100 Å, 5 μm; Dionex). After 4 min, the precolumn was connected with the separating nanocolumn Pepmap C18 (0.075 by 15 cm, 100 Å, 3 μm), mobile phase A was 2% CH_3_CN, 0.1% aqueous FA, and mobile phase B was 80% CH_3_CN, 20% H_2_O, 0.1% FA. The flow rate was constant at 300 nl min^−1^ and the mobile phase composition was as follows: 0% B for 4 min; linear increase over 31 min to 38% B to separate the tryptophan derivatives. Mass analyses were realized on an Orbitrap analyzer or in a linear trap.

## Additional information

**How to cite this article:** Benjdia, A. *et al*. The thiostrepton A tryptophan methyltransferase TsrM catalyses a cob(II)alamin-dependent methyl transfer reaction. *Nat. Commun.* 6:8377 doi: 10.1038/ncomms9377 (2015).

## Supplementary Material

Supplementary Information Supplementary Figures 1-11, Supplementary Tables 1-4, Supplementary Note 1 and Supplementary References
