# Supplementary material for: The thiostrepton A tryptophan methyltransferase TsrM catalyses a cob(II)alamin-dependent methyl transfer reaction
Source: Nat Commun. 2015 Oct 12;6:8377. doi: 10.1038/ncomms9377 (PMC4632189; doi:10.1038/ncomms9377)
Supplement: Supplementary Information — Supplementary Figures 1-11, Supplementary Tables 1-4, Supplementary Note 1 and Supplementary References [file ncomms9377-s1.pdf]

Supplementary Figure 1

A

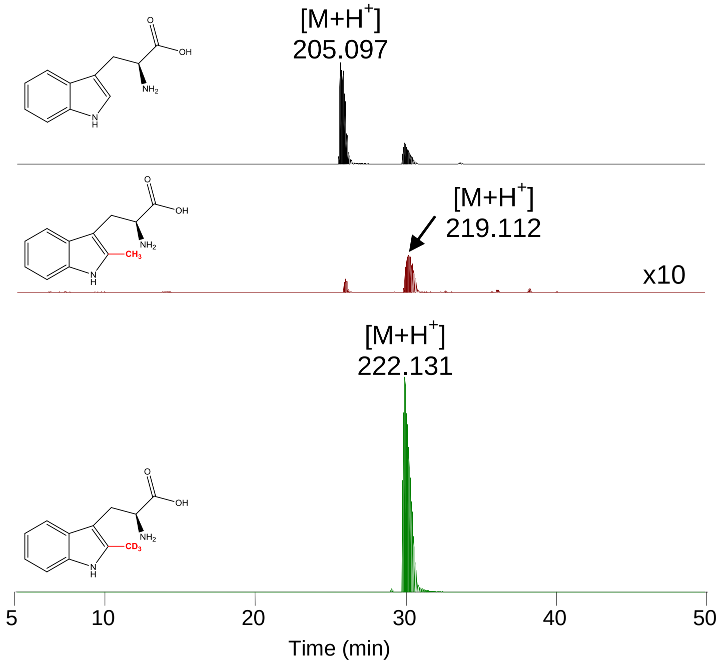

LC-MS analysis of TsrM incubated with Trp, *d3*-SAM and MeCbl. (**A**) Upper trace corresponds to *m/z* extracted-ion chromatogram of the Trp (m/z= 205.09), middle trace to *m/z* extracted-ion chromatogram of MeTrp (This trace has been expanded ten-times) (m/z= 219.112) and lower trace to *m/z* extracted-ion chromatogram of Me-(d_3)-Trp (m/z= 222.131).

Supplementary Figure 2

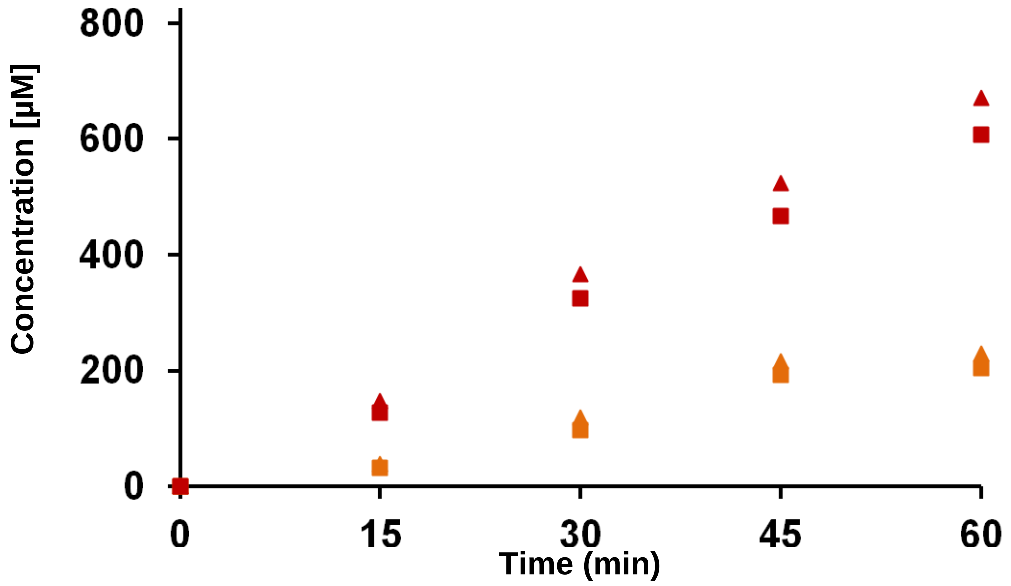

Time course for the production of 2-Me-Trp (■) and SAH (▲) by TsrM incubated under anaerobic conditions in the presence of 5 µM MeCbl (red symbols) or 1 µM MeCbl (orange symbols) in the presence of 1mM SAM, 1 mM Trp and 6 mM DTT.

Supplementary Figure 3

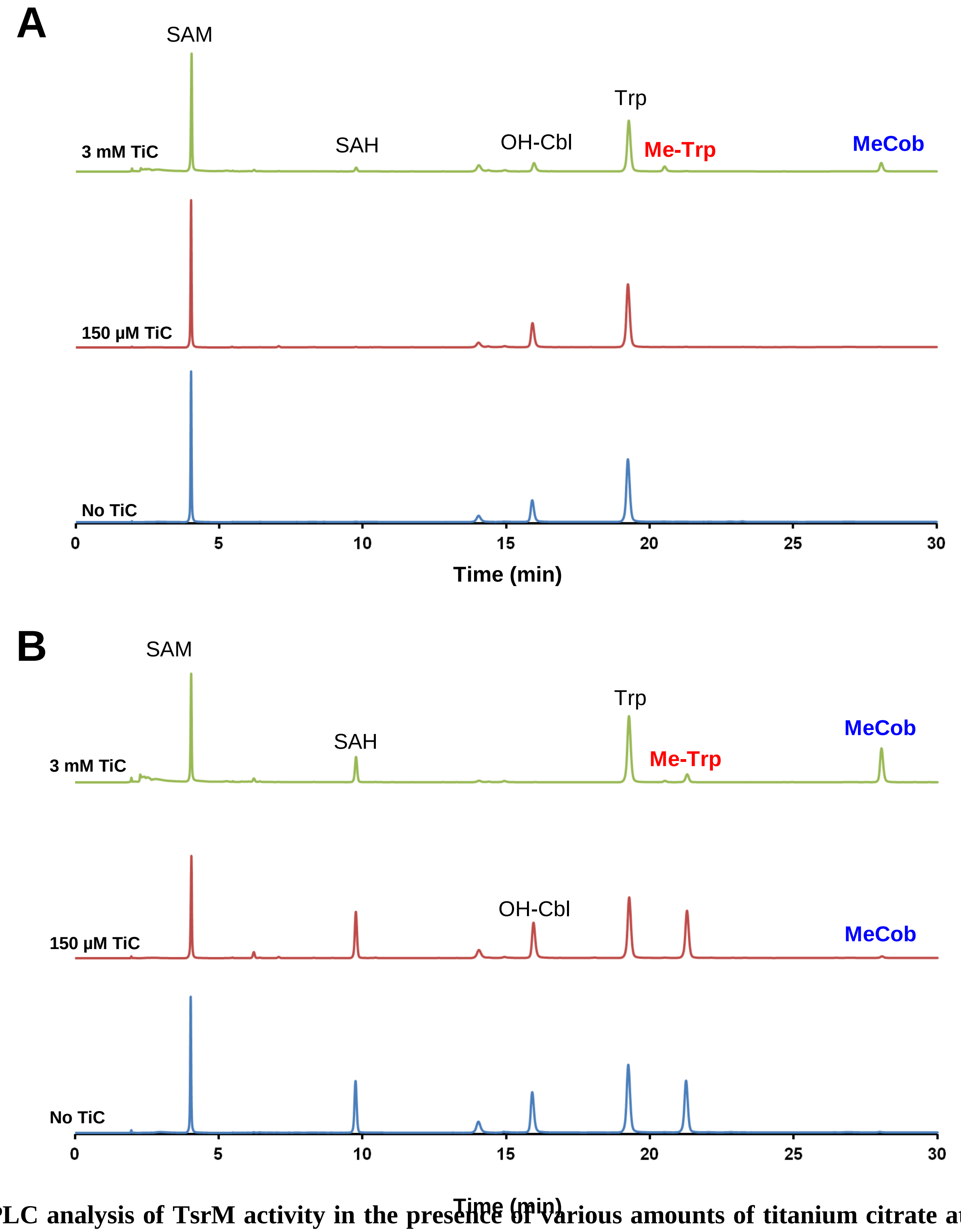

HPLC analysis of TsrM activity in the presence of various amounts of titanium citrate at T0 (A) and after 2 hours of reaction (B). TsrM (23 µM) was incubated under anaerobic conditions in the presence of DTT (3 mM), SAM (1 mM), Trp (1 mM) OHCbl (200 µM) and various amounts of titanium citrate (from top to bottom: 0, 150 µM or 3 mM). HPLC analysis was performed on a C18 column with UV detection at 278nm.

Supplementary Figure 4

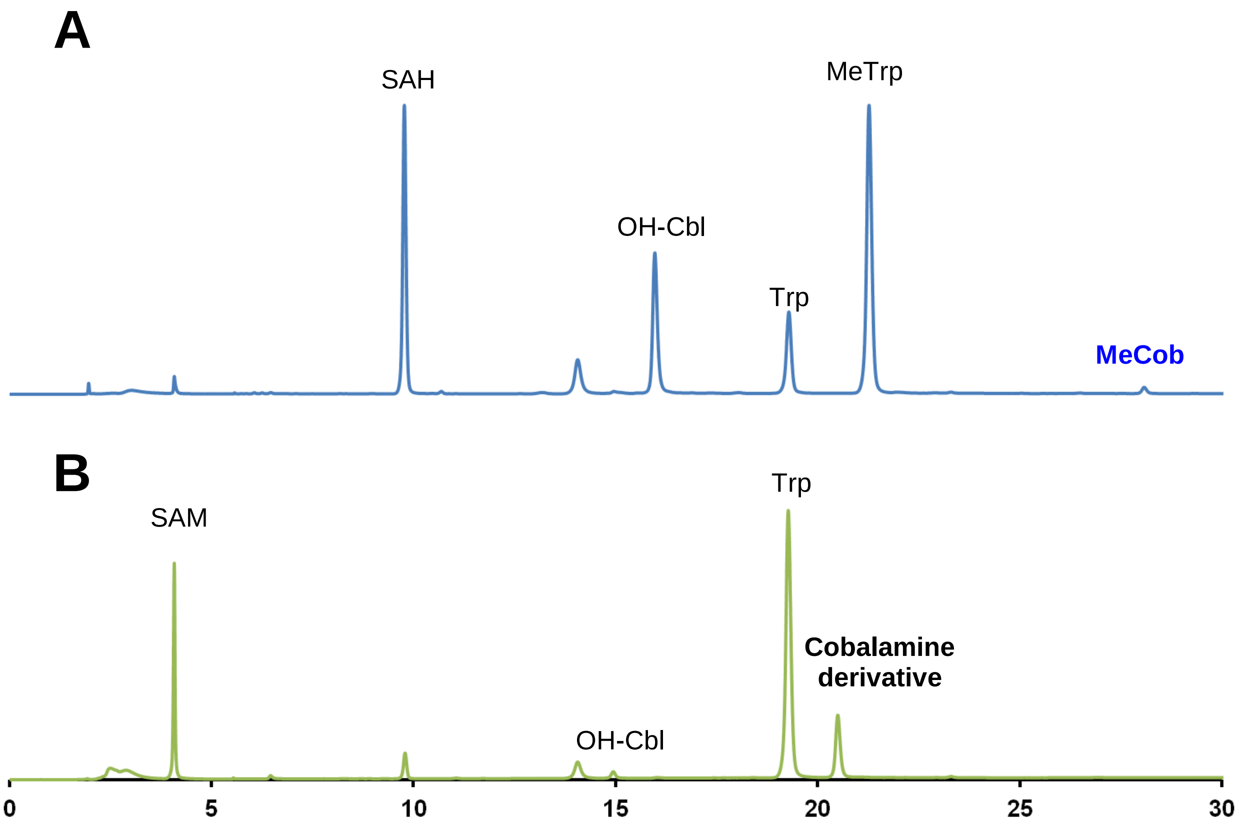

HPLC analysis of TsrM reaction and a control reaction with a 10-times excess of free iron and sulfur. (**A**) TsrM was incubated with DTT (3 mM), SAM (1 mM), Trp (1 mM) OHCbl (1 mM). (**B**) Control reaction with 30 mM DTT, 5mM Fe, 5mM Sulfur, SAM (1 mM), Trp (1 mM) OHCbl (1 mM). HPLC analysis was performed on a C18 column with UV detection at 278nm.

Supplementary Figure 5

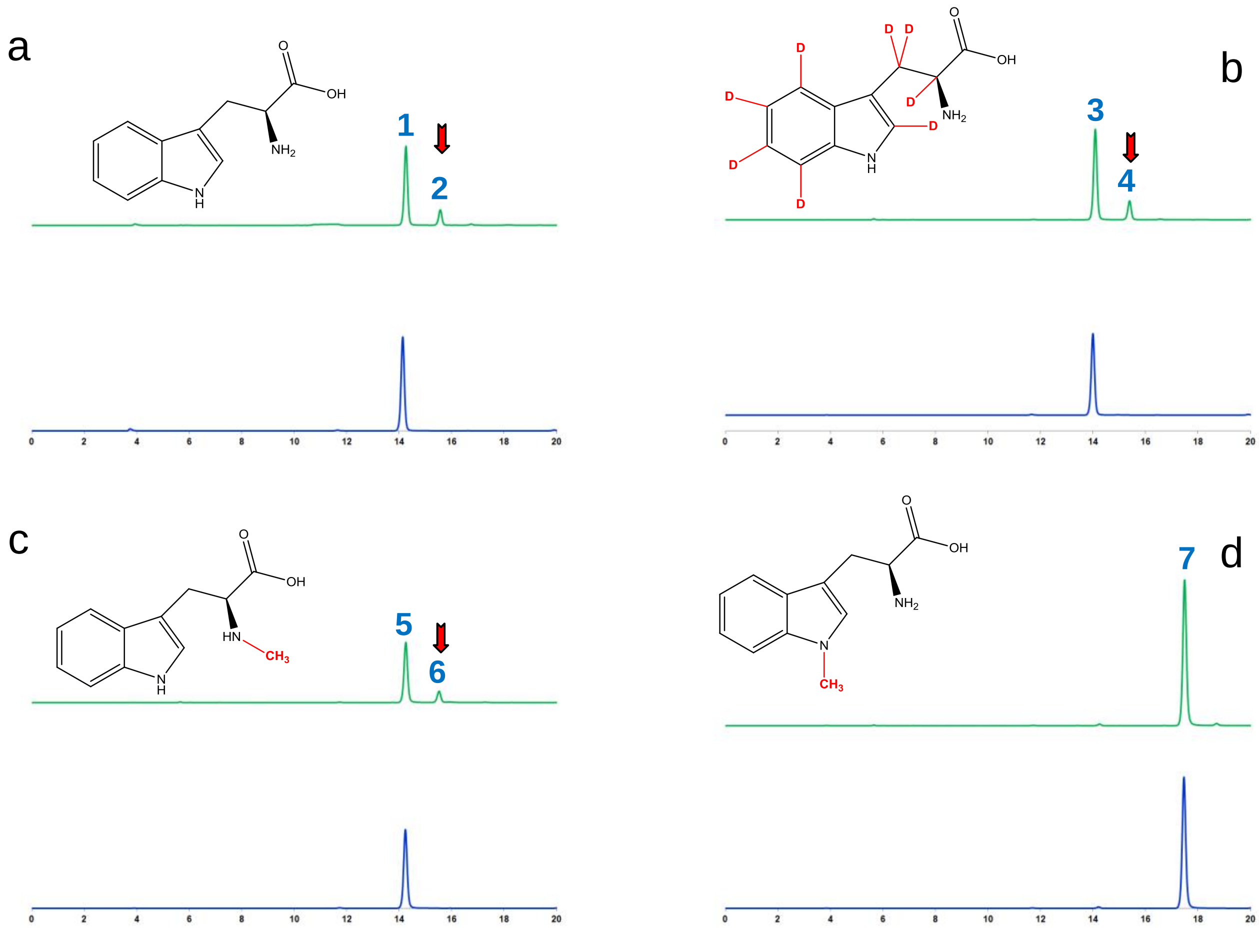

HPLC analysis of the TsrM reaction with: (a) Trp, (b) L-Trp (d_8, $^{15}N_2$), (c) N_α- CH_3-L-Trp or (d) 1-CH_3-Trp at T0 (lower traces) and T12H (upper traces). TsrM (20 µM) was incubated under anaerobic conditions in the presence of 6 mM DTT with MeCbl (500 µM) and the different substrates at 1 mM. Detection was performed by fluorescence (Ex/Em: 280/350) (see **Figs**. **S6-S9 & Table S1** for MS analyses).

Supplementary Figure 6

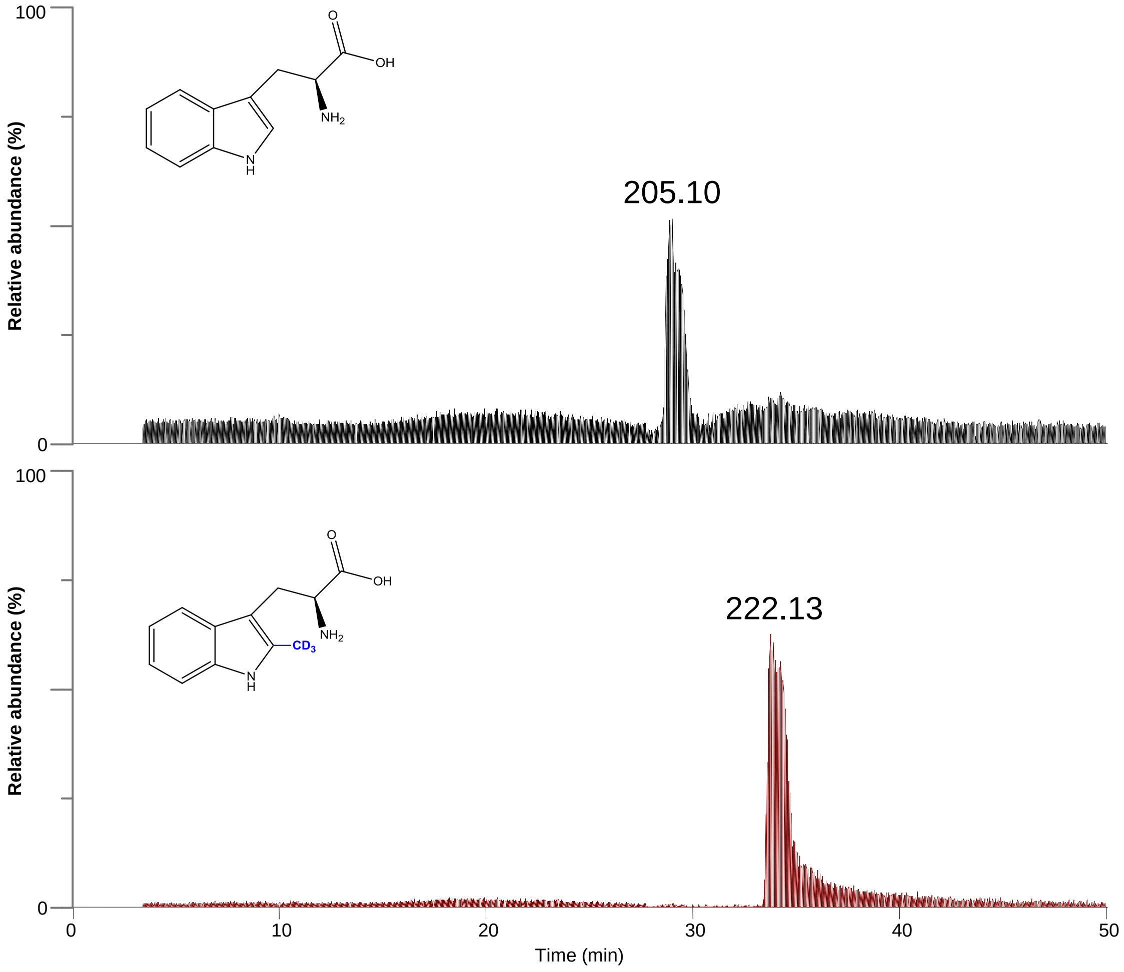

LC-MS analysis of TsrM incubated with Trp, *d*$_3$-SAM and MeCbl. (**a**) Upper trace corresponds to *m/z* extracted-ion chromatogram of the substrate (m/z= 205.09) and the lower trace to *m/z* extracted-ion chromatogram of CH_3-(d_3)-Trp (m/z= 222.131) (**see Table S1 for assignement**).

Supplementary Figure 7

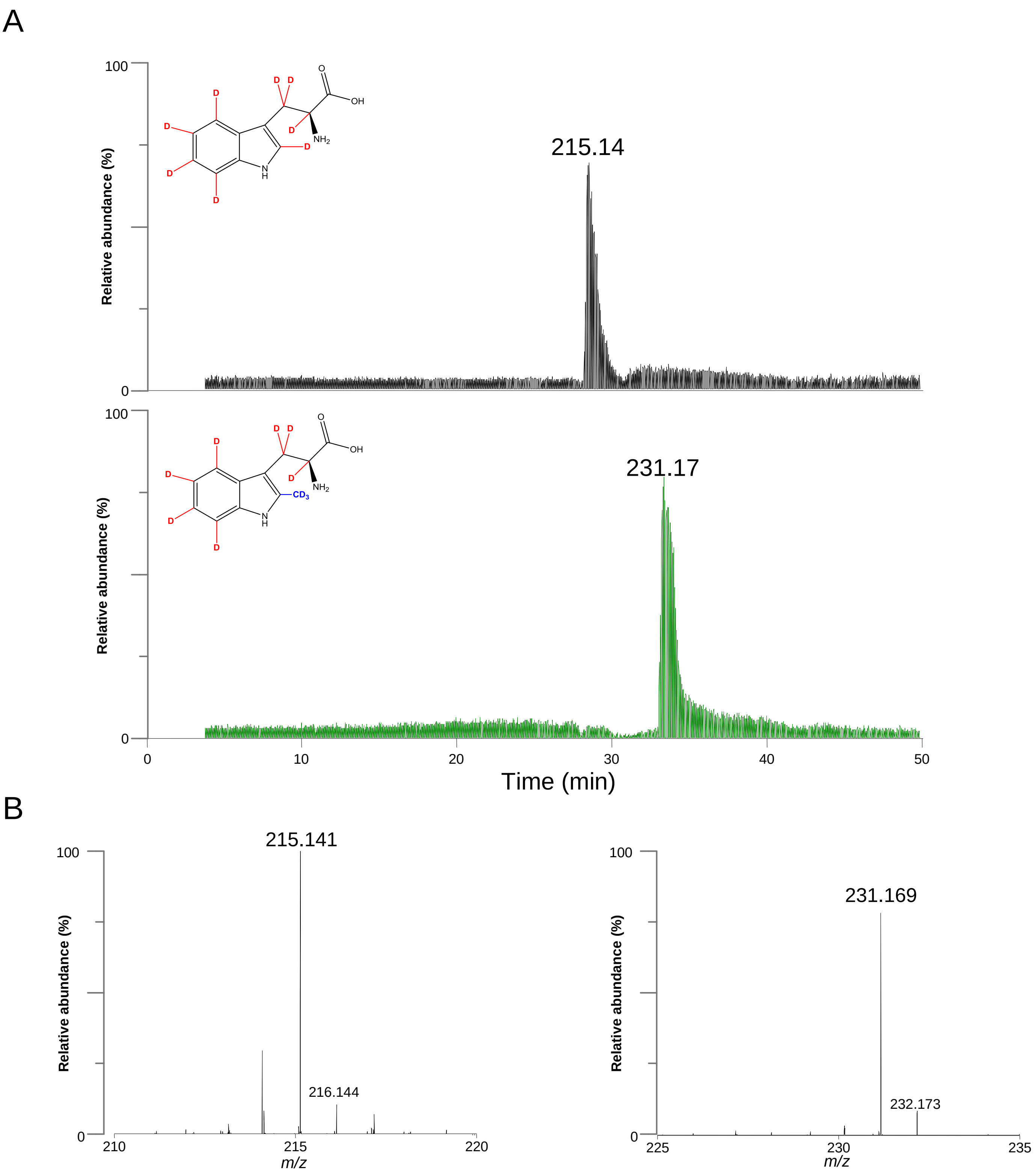

(A) LC-MS analysis of TsrM incubated with L-Trp (d_8, $^{15}N_2$), d_3-SAM and MeCbl. (**a**) Upper trace corresponds to *m/z* extracted-ion chromatogram of the substrate (m/z= 215.14) and lower trace to *m/z* extracted-ion chromatogram of CD3-Trp (d_{10}, $^{15}N_2$) (m/z= 231.17) (**see Table S1 for assignement**).
(B) Mass spectrometry analysis of the substrate and the product.

Supplementary Figure 8

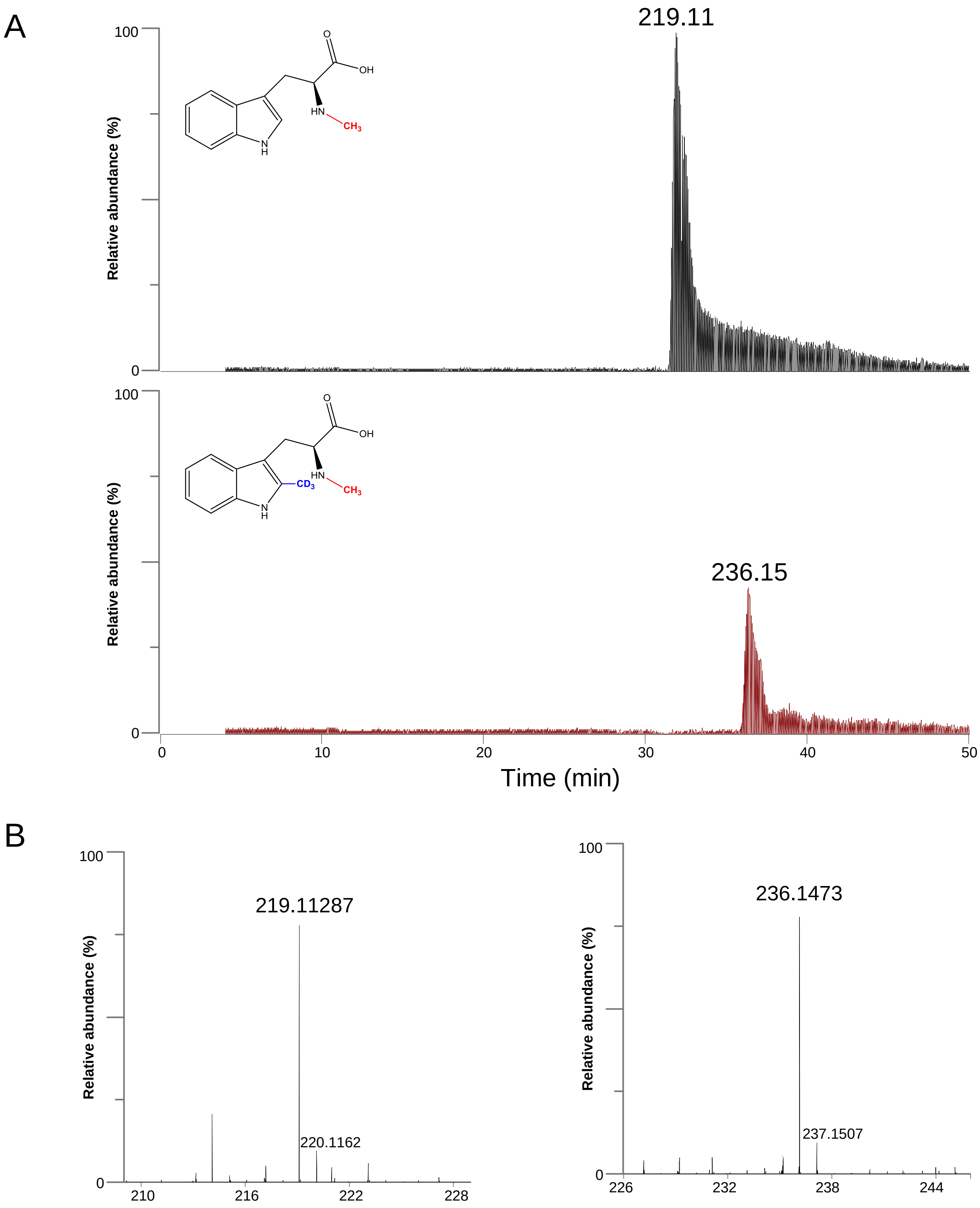

(A) LC-MS analysis of TsrM incubated with N_{α}**-**CH_3**-L-Trp,** d_3**-SAM and MeCbl.** Upper trace corresponds to *m/z* extracted-ion chromatogram of the substrate (m/z= 219.11) and lower trace to *m/z* extracted-ion chromatogram of dimethyltryptophan (m/z= 236.15) (**see Table S1 for assignment**).

(B) Mass Spectrometry analysis of the substrate and the product.

Supplementary Figure 9

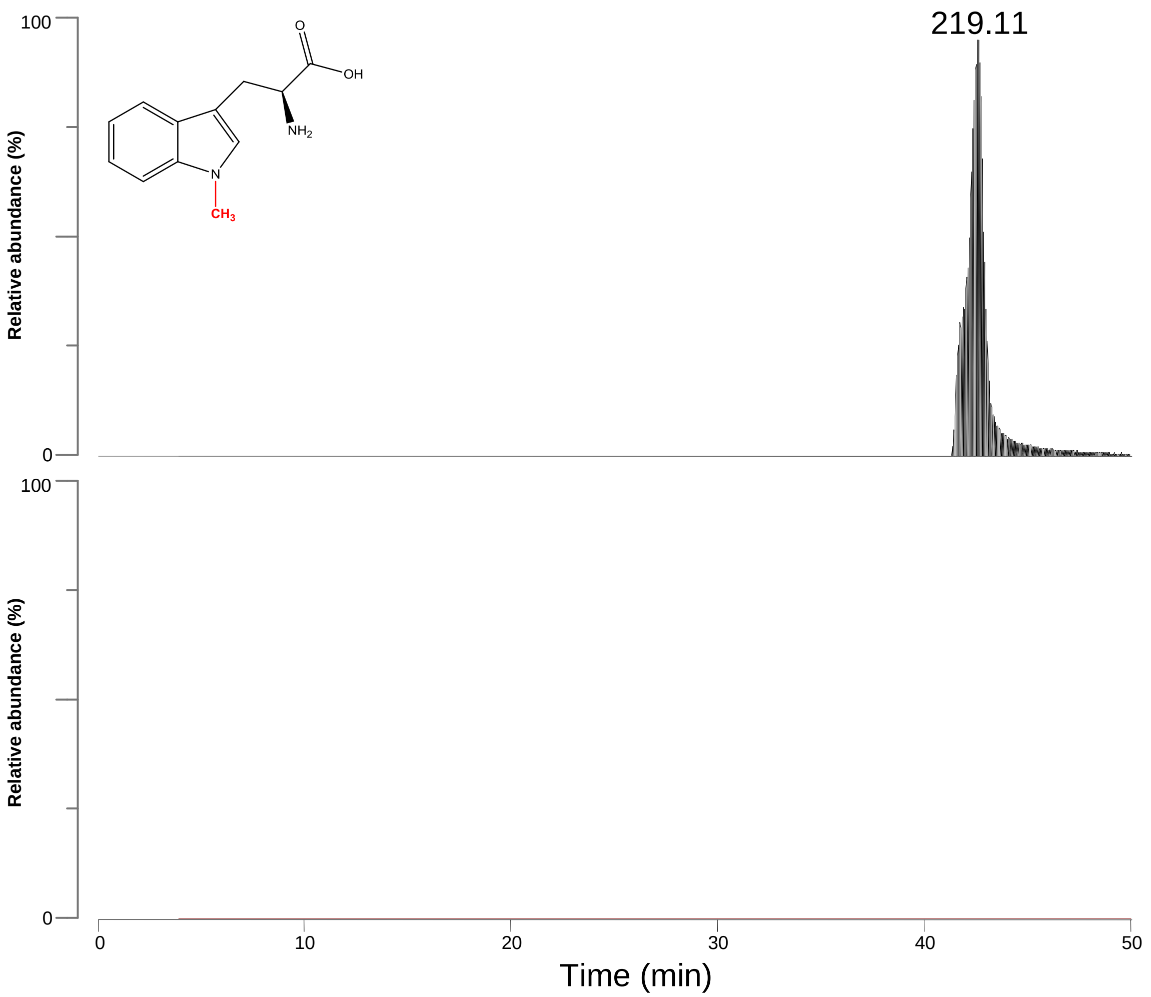

LC-MS analysis of TsrM incubated with NM$_1$-L-Trp, *d*$_3$-SAM and MeCbl. Upper trace corresponds to *m/z* extracted-ion chromatogram of the substrate (m/z= 219.11) and the lower trace to *m/z* to *m/z* extracted-ion chromatogram of dimethyltryptophan (m/z= 236.15) (**see Table S1 for assignment**).

Supplementary Figure 10

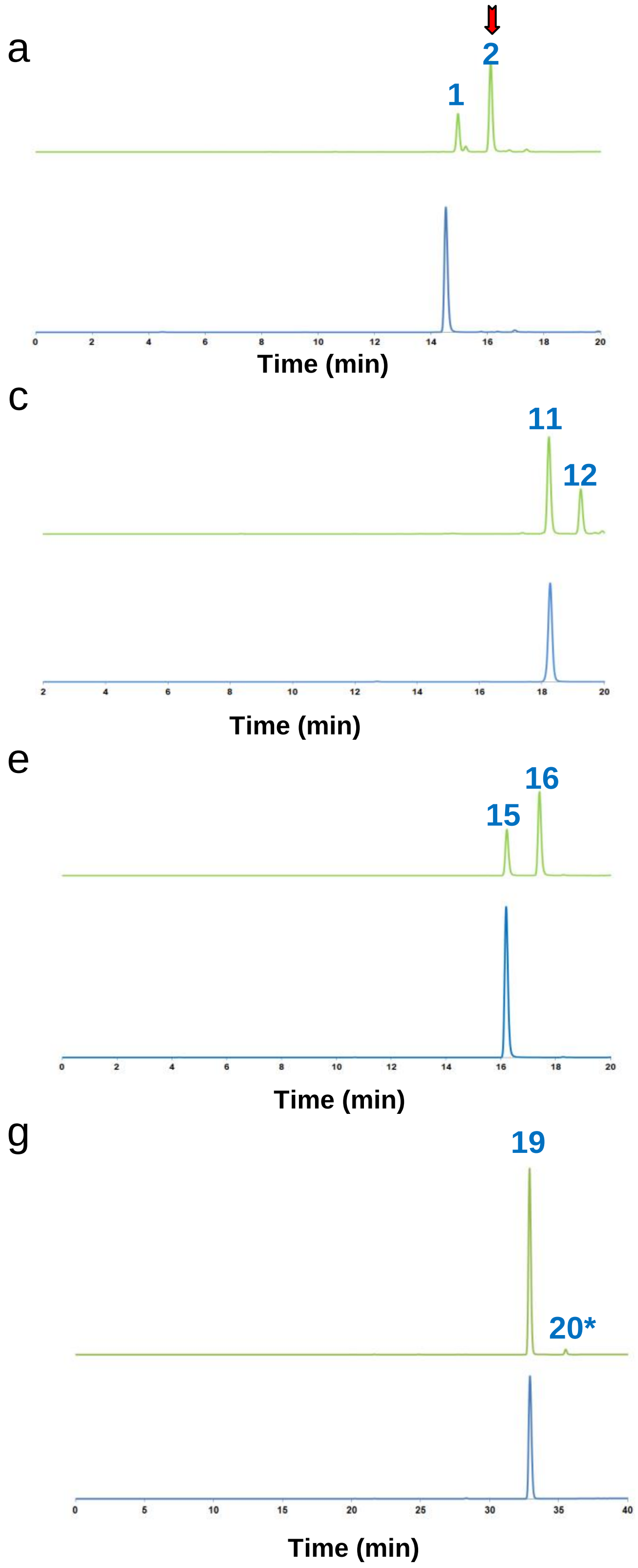

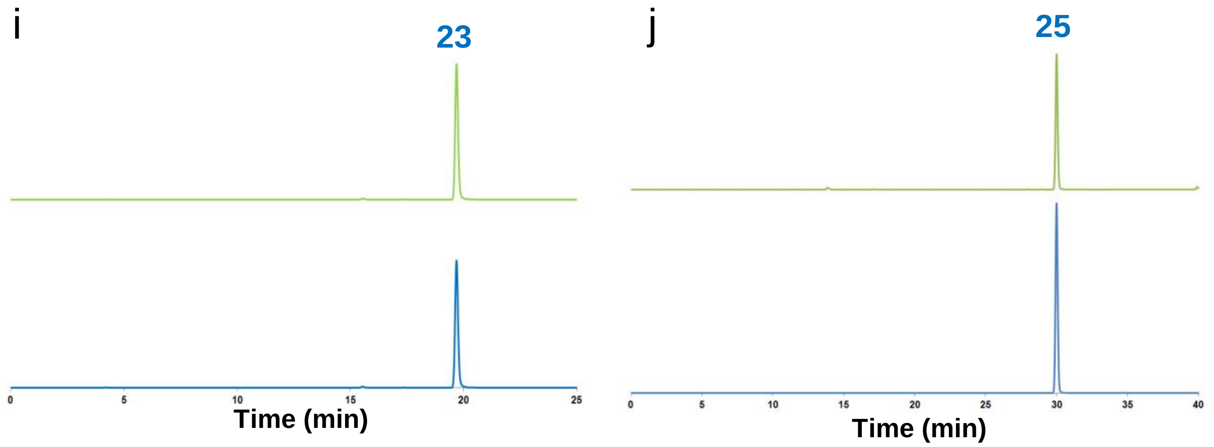

HPLC analysis coupled with fluorescence detection (Ex/Em: 280/350 m) of reactions of TsrM with tryptophan derivatives at T0 (blue traces) and T12H (green traces). Product numbering and characterization are described in Table S1.

TsrM (20 µM) was incubated under anaerobic conditions with DTT (6 mM), methylcobalamin (500 µM), d_3-SAM (1 mM) and 1 mM (**a**) L-tryptophan **1**, (**b**) D-tryptophan **9**, (**c**) 5-methyl-L-tryptophan **11**, (**d**) 5-hydroxy-L-tryptophan **13**, (**e**) 5-fluoro-L-tryptophan **15**, (**f**) 6-fluoro-D/L-tryptophan **17**,(**g**) 3-methyl-indole **19**, (**h**) serotonin **21,** (**i**) melatonin **23,** or (**j**) indole **25**.

Depending on the substrate, the corresponding methylated derivatives (**a**) 2-methyl(d_3)-L-tryptophan **2**, (**b**) 2-methyl(d_3)-D-tryptophan **10**, (**c**) 2-methyl(*d3*)-5-methyl-L-tryptophan **12**, (**d**) 2-methyl(d_3)-5-hydroxy-L-tryptophan **14**, (**e**) 2-methyl(d_3)-5-fluoro-L-tryptophan **16**, (**f**) 2-methyl(d_3)-6-fluoro-D/L-tryptophan **18,** (**g**) 2-methyl(d_3)-3 methyl-indole **20** or (**h**) 2-methyl(d_3)-serotonin **22** were formed (see **Figs**. **S11 & Table S2** for MS analyses)

The compound **20 was tentatively attributed to 2-methyl(d_3)-3 methyl-indole but its molecular weight could not be measured.*

Supplementary Figure 11

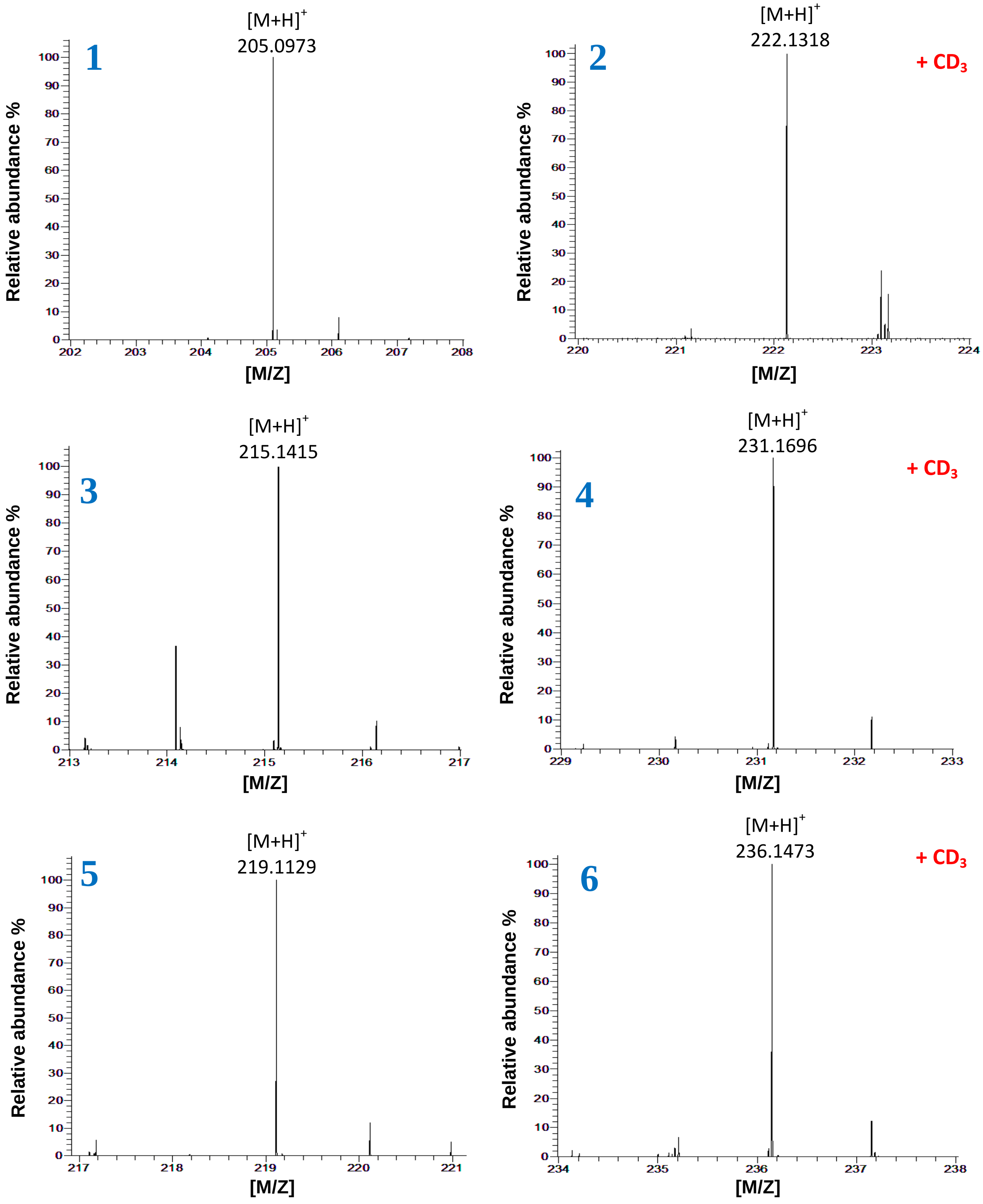

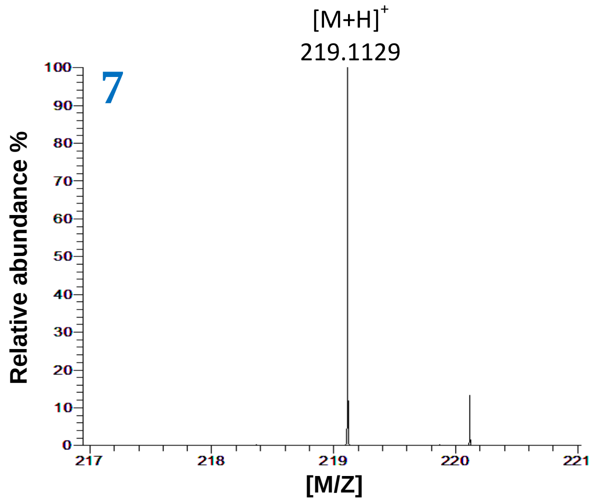
[M+H]+
219.1129
7
Relative abundance %
[M/Z]

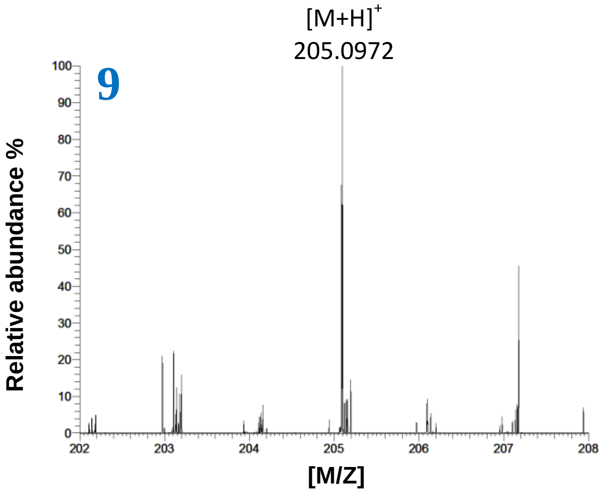
[M+H]+
205.0972
9
Relative abundance %
[M/Z]

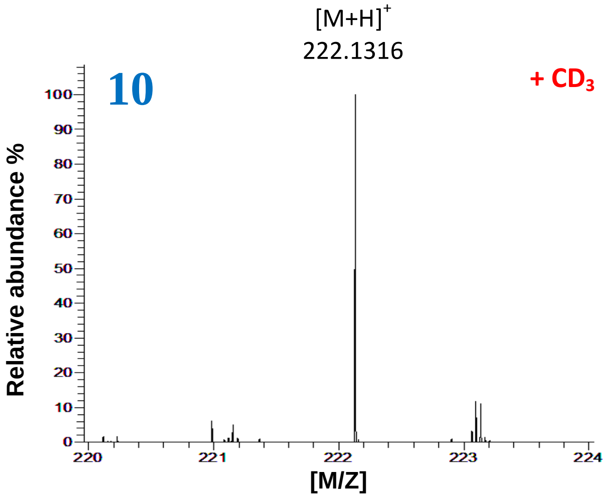
[M+H]+
222.1316
10
+ CD3
Relative abundance %
[M/Z]

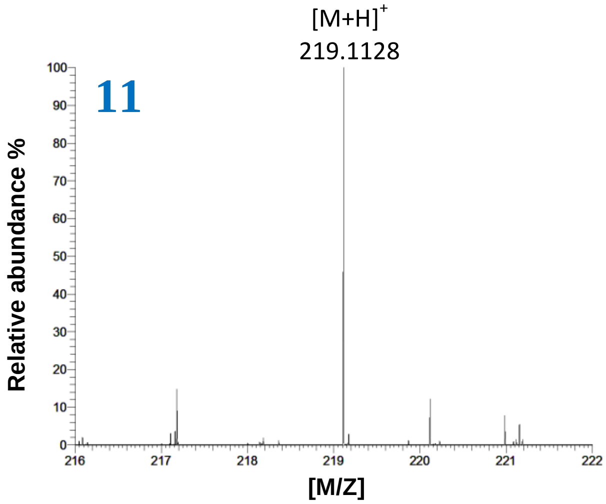
[M+H]+
219.1128
11
Relative abundance %
[M/Z]

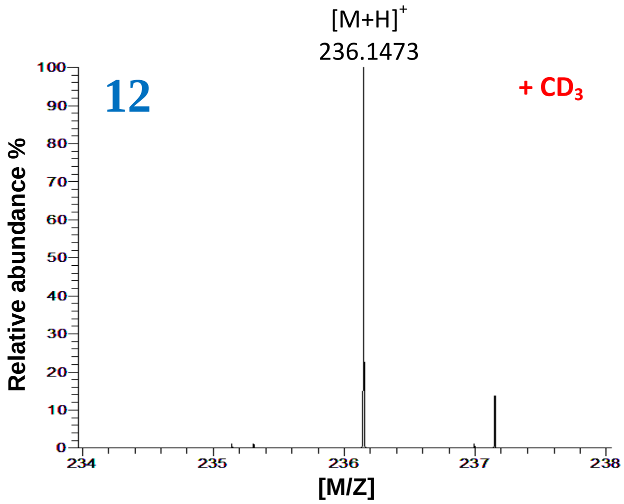
[M+H]+
236.1473
12
+ CD3
Relative abundance %
[M/Z]

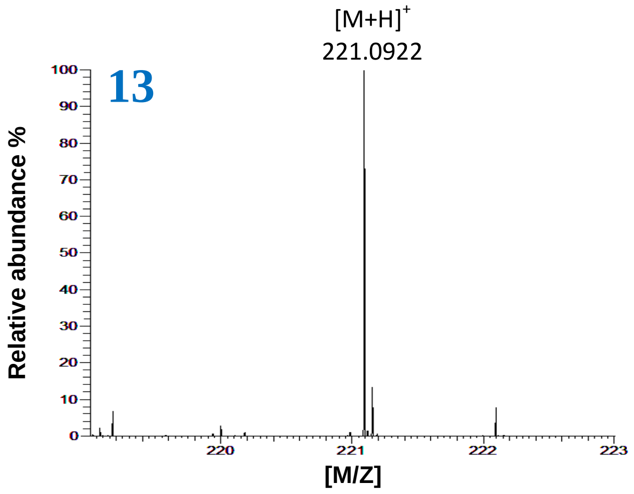
[M+H]+
221.0922
13
Relative abundance %
[M/Z]

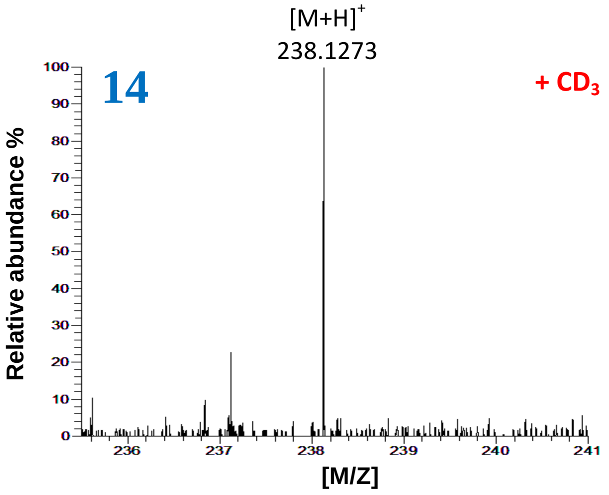
[M+H]+
238.1273
14
+ CD3
Relative abundance %
[M/Z]

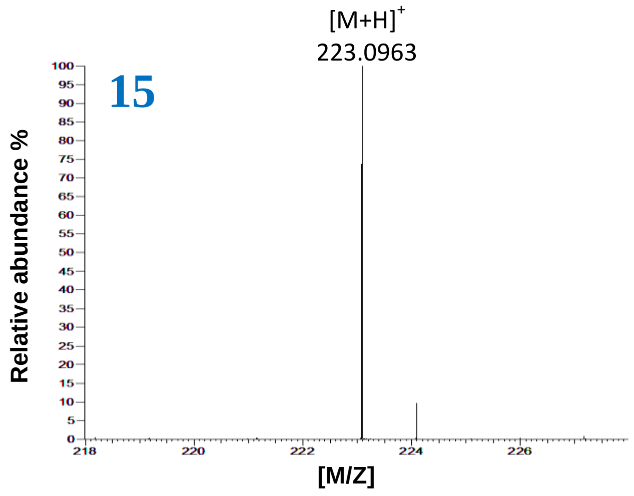
[M+H]+
223.0963
15
Relative abundance %
[M/Z]

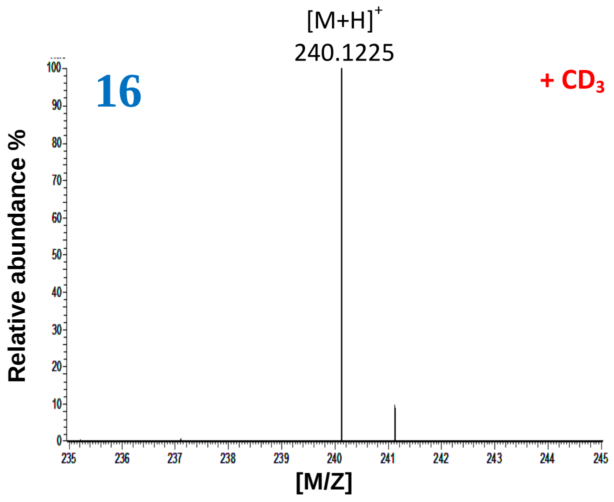
[M+H]+
240.1225
16
+ CD3
Relative abundance %
[M/Z]

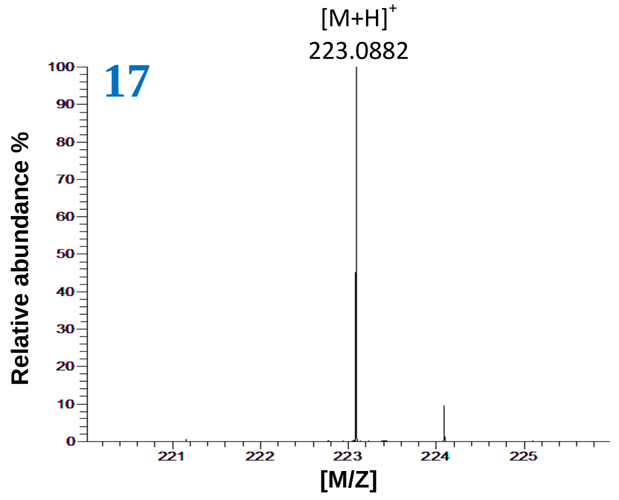
[M+H]+
223.0882
17
Relative abundance %
[M/Z]

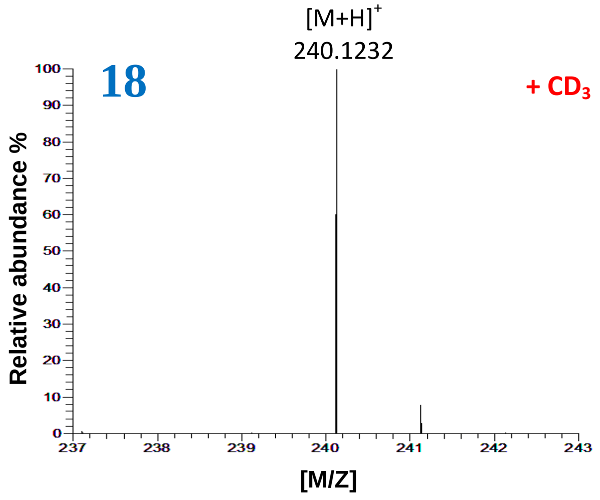
[M+H]+
240.1232
18
+ CD3
Relative abundance %
[M/Z]

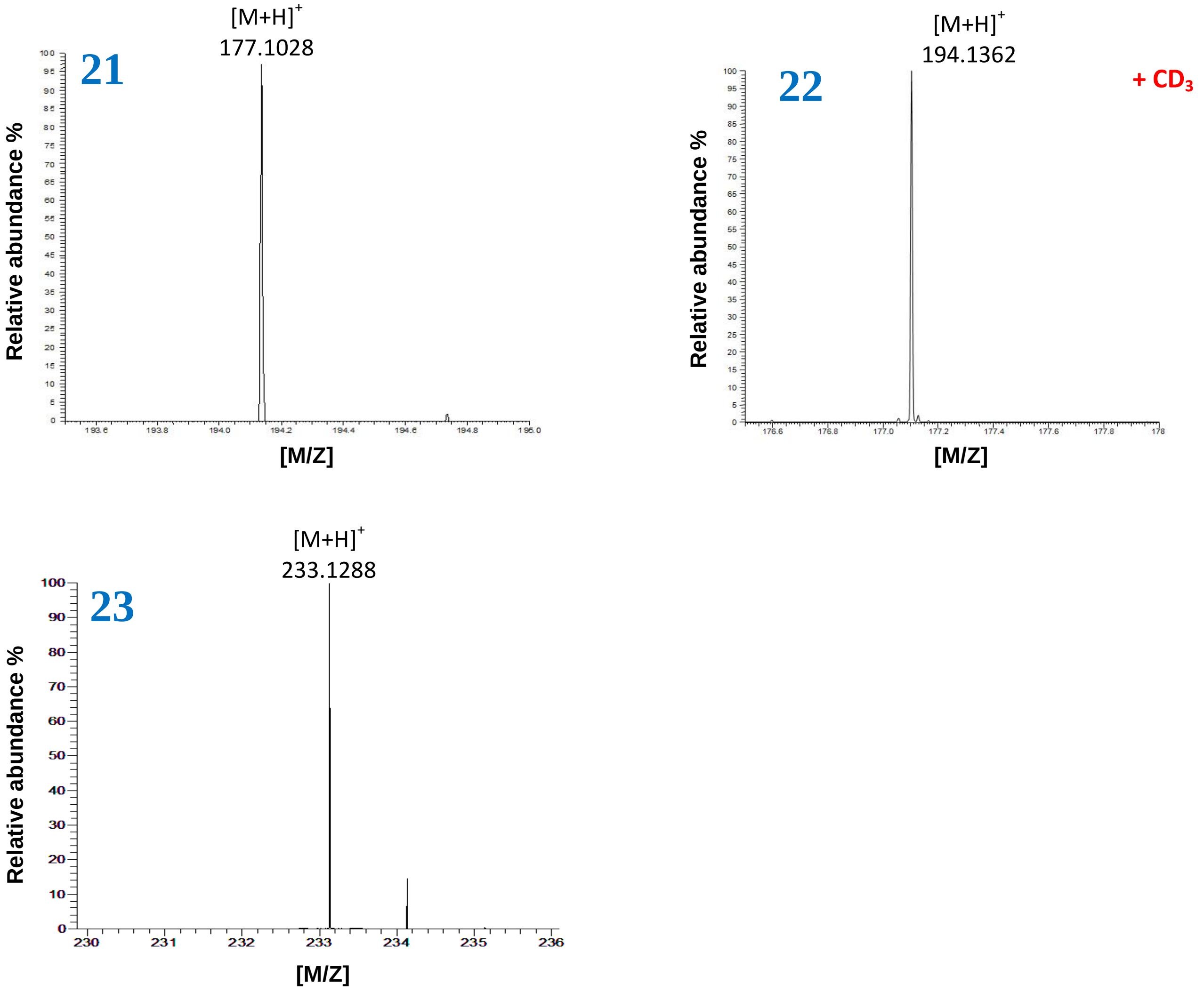

Mass spectrometric analysis of substrate and products formed by TsrM (see Tables S1&S2 for compound numbering and characterization). TsrM (20 µM) was incubated under anaerobic conditions with DTT (6 mM), MeCbl (500 µM), d_3-SAM (1 mM) and different substrates: (**1**) L-Trp, (**3**) L-Trp (d_8, $^{15}N_2$), (**5**) N_α-CH_3-L-Trp, (**7**) 1-CH_3-D/L-Trp, (**9**) D-Trp, (**11**) 5-CH_3-L-Trp, (**13**) 5-Hydroxy-L-Trp, (**15**) 5-Fluoro-L-Trp, (**17**) 6-Fluoro-DL-Trp, (**21**) serotonin, (**23**) melatonin and formed the corresponding CD_3

Methylated products : (**2**) 2-CD_3-L-tryptophan, (**4**) 2-CD_3-L-Trp (D8, ^{15}N2), (**6**) 2-CD_3-N_α-CH_3-L-Trp, (**10**) 2-CD_3-D-Trp, (**12**) 2-CD_3-5-CH_3-L-Trp, (**14**) 2-CD_3-5-hydroxy-L-Trp, (**16**) 2-CH_3-5-Fluoro-L-Trp, (**l8**) 2-CD_3-6-Fluoro-D/L-Trp, (**22**) 2-CH_3-serotonin.

Supplementary Table 1: Isotopically labeled and methylated Trp derivatives used as TsrM substrates and the corresponding products formed

compound	Name	Structure	Formula	HPLC Retention time (Min)	Relative activity*	[M+H]+	$[M+H]^+$ experimental
**1**	L-Tryptophan	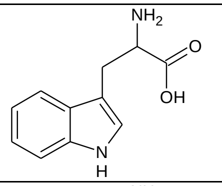	$C_{11}H_{12}N_2O_2$	14.6		205.09715	205.0973
**2**	2-Methyl(D_3)-L- Tryptophan	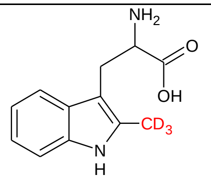	$C_{12}H_{11}D_3N_2O_2$	15.8	**100**	222.13163	222.1318
**3**	L-tryptophan (D8, $^{15}N_2$)	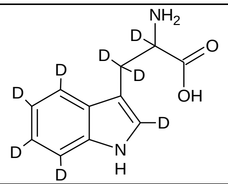	$C_{11}D_8H_4N_2O_2$	14.1		215.14117	215.1415
**4**	2-Methyl(D_3)-L-tryptophan (D8, $^{15}N_2$)	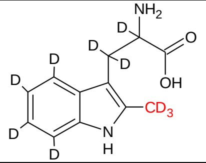	$C_{12}H_4D_{10}N_2O_2$	15.4	**100**	231.16917	231.1696
**5**	N_α-methyl-L-tryptophan	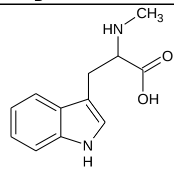	$C_{12}H_{14}N_2O_2$	14.25		219.11280	219.1129
**6**	2-Methyl(D_3)-N_α-methyl-L-tryptophan	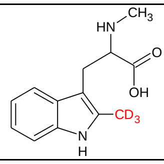	$C_{13}H_{13}D_3N_2O_2$	15.55	**80**	236.14728	236.1473

**7**	1-Methyl-DL-tryptophan	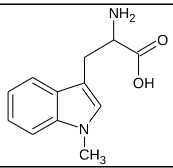	$C_{12}H_{14}N_2O_2$	17.4		219.11280	219.1129
**8**	1-Methyl-DL-tryptophan	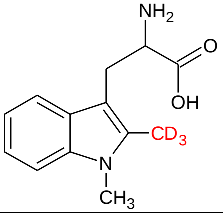	$C_{13}H_{13}D_3N_2O_2$	*nd*	*nd*	236.14728	*nd*

Structure, formula, mass retention time by HPLC (see the Methods section) and activity are indicated. *nd*: not detected. *The relative activity was measured as the amount of SAH produced compared to the amount obtained in reaction with Trp used as substrate.

Supplementary Table 2: Tryptophan derivatives used as potential TsrM substrates and the corresponding formed products

Compound	Name	Structure	Formula	Retention time (min)	Activity*	$[M+H]^+$ theoretical	$[M+H]^+$ experimental
**9**	D-Tryptophan	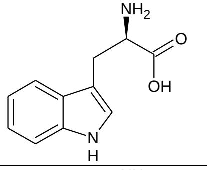	$C_{11}H_{12}N_2O_2$	15.1		205.09715	205.0972
**10**	2-Methyl(D_3)-D- Tryptophan	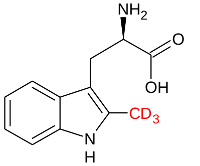	$C_{12}H_{11}D_3N_2O_2$	16.4	**50**	222.13163	222.1316
**11**	5-Methyl-L-Tryptophan	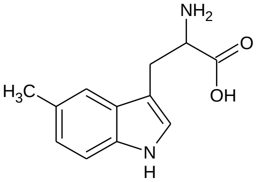	$C_{12}H_{14}N_2O_2$	18.2		219.11280	219.1128
**12**	2,5-dimethyl-L-Tryptophan	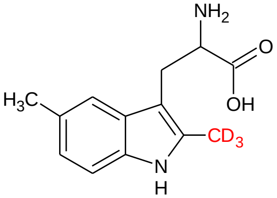	$C_{13}H_{13}D_3N_2O_2$	19.2	**97**	236.14728	236.1473
**13**	5-Hydroxy-L-Tryptophan	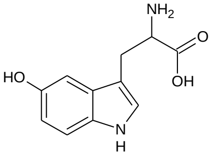	$C_{11}H_{12}N_2O_3$	10.4		221.09207	221.0922
**14**	2-Methyl(D_3)-5-Hydroxy-L-Tryptophan	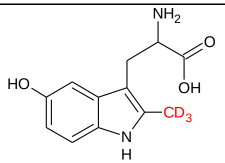	$C_{12}H_{11}D_3N_2O_3$	11.9	**75**	238.12655	238.1273

**15**	5-Fluoro-L-Tryptophan	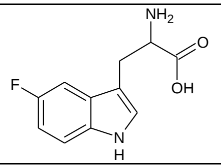	$C_{11}H_{11}FN_2O_2$	16.2		223.08773	223.0963
**16**	2-Methyl(D_3)-5-Fluoro-L-Tryptophan	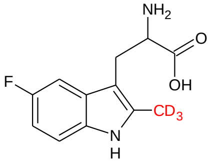	$C_{12}H_{10}D_3FN_2O_2$	17.4	**87**	240.12221	240.1225
**17**	6-Fluoro-DL-Tryptophan	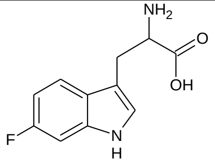	$C_{11}H_{11}FN_2O_2$	16.3		223.08773	223.0882
**18**	2-Methyl(D_3)-6-Fluoro-DL-Tryptophan	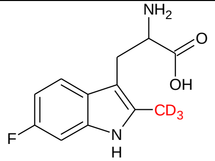	$C_{12}H_{10}D_3FN_2O_2$	17.6	**39**	240.12221	240.1232
**19**	3-Methyl-indole	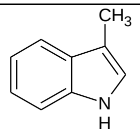	C_9H_9N	32.9		132.08078	
**20**	2-Methyl(D_3)-3-Methyl-indole	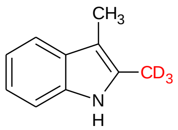	$C_{10}H_8D_3N$	35.5	**21**	149.11526	
**21**	Serotonin	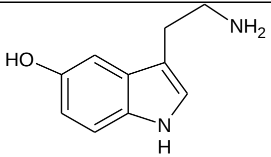	$C_{10}H_{12}N_2O$	10		177.10224	177.1025
**22**	2-Methyl(D_3)-Serotonin	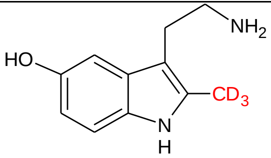	$C_{11}H_{11}D_3N_2O$	11.8	**26**	194.113672	194.1362

**23**	Melatonin	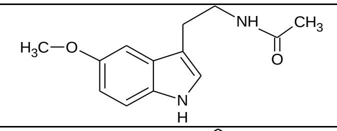	$C_{13}H_{16}N_2O_2$	19.7		233.12845	233.1288
**24**	2-Methyl(D_3)-Melatonin	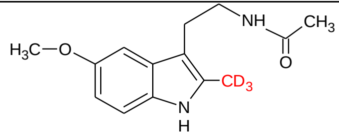	$C_{14}H_{15}D_3N_2O_2$	*nd*	*nd*	250.16293	*nd*
**25**	indole	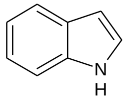	C_8H_7N	27.6		118.0651	
**26**	2-methyl(D_3)-indole	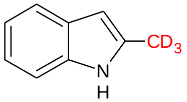	$C_9H_6D_3N$	*nd*	*nd*	135.09961	*nd*

Structure, formula, mass retention time by HPLC (see the Methods section) and activity are indicated. *nd*: not detected. **The relative activity was measured as the amount of SAH produced compared to the amount obtained in reaction with Trp used as substrate.*

Supplementary Table 3. **Key geometrical parameters in the transition state structure for the methyl radical addition to ethene and ethyne using different levels of theory.**

	$C_{\bullet CH3}$-C_{C2H4} (Å)	$[C\text{-}C]_{C2H4}$ (Å)	$C_{\bullet CH3}$-$[C\text{-}C]_{C2H4}$ (°)
B3LYP/6-31G**	2.36	1.36	109.9
M06-2X/6-31G**	2.27	1.35	109.4
CCSD(T)/6-311G(d,p) [a]	2.289	1.368	109.1
	$C_{\bullet CH3}$-C_{C2H2} (Å)	$[C\text{-}C]_{C2H2}$ (Å)	$C_{\bullet CH3}$-$[C\text{-}C]_{C2H2}$ (°)
M06-2X/6-31G**	2.24	1.22	115.0
CCSD(T)/6-311G(d,p)[a]	2.241	1.235	115.2

[a]from ref (3)

Supplementary Table 4. **Energy barriers of methyl addition relative to the reactants' are calculated at the M06-2X/cc-pvtz(-f) level of theory from geometries obtained with M06-2X/6-31G**.** Energies are given in kcal/mol. Calculations for addition to C5 and C7 led to similar energy barriers when compared to C6 and C4, respectively (not shown).

	Reactants	Transition State	Product
$\bullet CH_3$ on C2-Trp	0	6.4	-25.6
$\bullet CH_3$ on C4-Trp	0	7.8	-19.5
$\bullet CH_3$ on C5-Trp	0	10.4	-12.2
$\bullet CH_3$ on C6-Trp	0	9.5	-15.5
$\bullet CH_3$ on C7-Trp	0	8.5	-19.0

Supplementary Note 1

Quantum calculations.

The Jaguar program included in the Schrödinger suite was employed for all quantum calculations (1).

Electrostatic potential maps

We geometry-optimized Trp, 5-Me-Trp, 5-OH-Trp and 5-F-Trp molecules at the B3LYP/6-31G** level of theory. We generated the electrostatic potential that was then mapped to the molecular electron density surface which was created using an isovalue of 0.001 au (Figure 2B).

•CH3 addition

Given the absence of a TsrM crystal structure, we modeled a methyl radical approaching in a plane parallel to the one defined by the tryptophan rings. The tryptophan amino acid moiety was set to neutral (-NH2, -COOH) because we performed the calculations in vacuo. We were aware that solvent effects might be important in this type of reaction (2), however, with no available structural information we decided to compare the methyl radical addition to the C2, C4, C5, C6 and C7 atoms in the gas phase. Results have been reported in the literature for the study of •CH3 addition to ethene or ethyne using the standard B3LYP functional and higher levels of theory (3). We tested the higher level M06-2X functional (4, 5) with the 6-31G** basis set (Supplementary Table 3). Since better results were obtained with this functional, it was employed for the transition state searches; all minima and transition states were checked with a frequency analysis. The C•CH3-CTrp distance was initially scanned from 3.0 to 1.5 Å, optimizing the geometry at each distance at the B3LYP/6-31G** level, and then the transition state for the radical addition was located and optimized with M06-2X/6-31G**. A larger basis set, cc-pvtz(-f) was employed for single point energies of the reactants, transition state and product. Similar calculations to model the addition of methyl radical to the C2 atom in 5OH-, 5F-, 5Me- and NMe-tryptophan were also performed.

Supplementary References

1 - Jaguar, version 8.2, Schrödinger, Inc., New York, NY, 2013.

2 - Lalevée J, Allonas X, Fouassier JP, Rinaldi D, Ruiz Lopez MF, Rivail JL (2005) Solvent effect on the radical addition reaction to double bond: experimental and quantum chemical investigations. *Chem Phys Letts* 415:202-205.

3 - Gómez-Balderas R, Coote ML, Henry DJ, Radom L (2004) Reliable theoretical procedures for calculating the rate of methyl radical addition to carbon-carbon double and triple bonds. *J Phys Chem A* 108:2874-2883.

4 - Zhao Y, Truhlar DG (2008) The M06 suite of density functionals for main group thermochemistry, thermochemical kinetics, noncovalent interactions, excited states, and transition elements: two new functionals and systematic testing of four M06-class functionals and 12 other functionals. *Theor Chem Acc* 120:215-241.

5 - Zhao Y, Truhlar DG (2008) How well can new-generation density functionals describe the energetics of bond-dissociation reactions producing radicals? *J Phys Chem A* 112:1095-1099.
